# Oxidant therapy improves adipogenic differentiation of adipose-derived stem cells in human wound healing

**DOI:** 10.1186/s13287-021-02336-3

**Published:** 2021-05-10

**Authors:** Christian Ploner, Tina Rauchenwald, Catherine E. Connolly, Karin Joehrer, Johannes Rainer, Christof Seifarth, Martin Hermann, Markus Nagl, Susanne Lobenwein, Doris Wilflingseder, Giuseppe Cappellano, Evi M. Morandi, Gerhard Pierer

**Affiliations:** 1 Department of Plastic, Reconstructive and Aesthetic Surgery, Medical University of Innsbruck, Anichstrasse 35, 6020 Innsbruck, Austria; 2 Tyrolean Cancer Research Institute, Innsbruck, Austria; 3 Institute for Biomedicine, Eurac Research, Affiliated Institute of the University of Lübeck, Bolzano, Italy; 4 Department of Ophthalmology, Medical University of Innsbruck, Innsbruck, Austria; 5 Department of Anesthesiology and Critical Care Medicine, Medical University of Innsbruck, Innsbruck, Austria; 6 Institute of Hygiene and Medical Microbiology, Medical University of Innsbruck, Innsbruck, Austria; 7 Center for Translational Research on Autoimmune and Allergic Disease (CAAD), Interdisciplinary Research Center of Autoimmune Diseases (IRCAD), Department of Health Sciences, Università del Piemonte Orientale, Novara, Italy

**Keywords:** Granulation tissue, Adipose-derived stem cells, Wound healing, Adipogenesis, Macrophage polarization

## Abstract

**Background:**

Adipose-derived stem cells (ASC) and adipocytes are involved in numerous physiological and pathophysiological conditions, which have been extensively described in subcutaneous and visceral fat depots over the past two decades. However, much less is known about ASC and adipocytes outside classical fat tissue depots and their necessity in tissue remodeling after injury. Therefore, we investigated the etiology of adipocytes in human granulation tissue and define their possible role wound healing.

**Methods:**

Identification of human wound tissue adipocytes was determined by immunohistochemical staining of granulation tissue sections from patients undergoing surgical debridement. Stromal cell fractions from granulation tissue and subcutaneous fat tissue were generated by collagenase type II-based protocols. Pro- and anti-inflammatory wound bed conditions were mimicked by THP1- and CD14^+^ monocyte-derived macrophage models in vitro. Effects of macrophage secretome on ASC differentiation and metabolism were determined by immunoblotting, flow cytometry, and microscopy assessing early and late adipocyte differentiation states. Functional rescuing experiments were conducted by lentiviral transduction of wildtype PPARG, IL1RA, and N-chlorotaurine (NCT) treatment.

**Results:**

Single and clustered adipocyte populations were detected in 11 out of 13 granulation tissue specimens and single-cell suspensions from granulation tissue showed adipogenic differentiation potential. Pro-inflammatory signaling by IFNG/LPS-stimulated macrophages (M (IFNG/LPS)) inhibited the maturation of lipid droplets in differentiated ASC. In contrast, anti-inflammatory IL4/IL13-activated macrophages (M (IL4/IL13)) revealed minor effects on adipocyte development. The M (IFNG/LPS)-induced phenotype was associated with a switch from endogenous fatty acid synthesis to glycolysis-dominated cell metabolism and increased pro-inflammatory cytokine production. Impaired adipogenesis was associated with increased, but seemingly non-functional, CEBPB levels, which failed to induce downstream PPARG and CEBPA. Neither transgenic PPARG overexpression, nor inhibition of IL1B was sufficient to rescue the anti-adipogenic effects induced by IFNG/LPS-activated macrophages. Instead, macrophage co-treatment during stimulation with NCT, a mild oxidant produced by activated granulocytes present in human wounds in vivo, significantly attenuated the anti-adipogenic effects.

**Conclusions:**

In conclusion, the appearance of adipocytes in wound tissue indicates a prevailing anti-inflammatory environment that could be promoted by NCT treatment and may be associated with improved healing outcomes.

**Graphical abstract:**

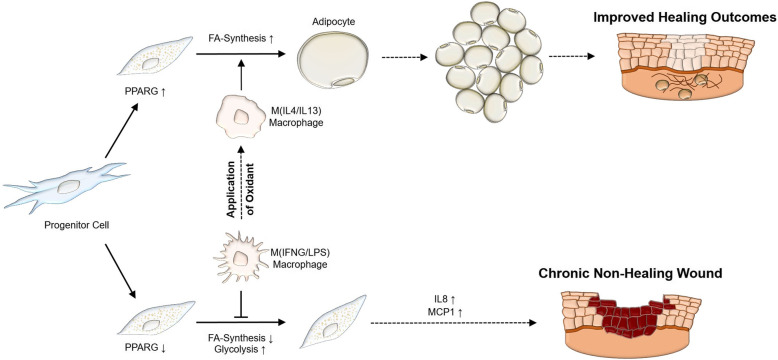

**Supplementary Information:**

The online version contains supplementary material available at 10.1186/s13287-021-02336-3.

## Background

Wound healing requires the activation and interaction of a variety of cell types in a highly orchestrated manner. Aside from the classical key players in wound healing, such as macrophages, endothelial cells, and (myo-)fibroblasts, emerging research has identified adipocytes as a novel participant [1]. In 2013, Schmidt and colleagues showed that adipocytes repopulated wound healing tissue in a murine wound model and contributed to fibroblast recruitment and extracellular matrix deposition [2]. In this study, lack of adipocytes resulted in increased wound bed size and reduced mechanical resistance of the de novo developed scar tissue [2]. In a human setting, autologous fat grafting enhanced healing outcomes of therapy-resistant chronic wounds of varying etiologies [3]. Similarly, injectable formulations of adipose-derived stem cells (ASC) promise therapeutic and regenerative capacity in a range of tissue and disease settings [4, 5]. These findings underline the significance of adipose tissue and its main cellular constituents for wound healing and suggest their role as highly dynamic and plastic participants in inflammatory signaling environments.

In human fat tissue, adipocytes repopulate from multipotent ASC ancestors, which undergo commitment to the adipocyte lineage (preadipocytes) and differentiate into unilocular lipid-laden adipocytes. This process is strictly controlled by adipogenic stimuli, which induce early adipogenesis initiating transcription factors of the CCAAT/enhancer-binding protein family, i.e., CEBPB and CEBPD. Finally, these transcription factors are replaced by key regulators of adipocyte differentiation, i.e., peroxisome proliferator-activated receptor gamma (PPARG) and CEBPA, which mutually induce the expression of each other and cooperate to activate genes controlling terminal differentiation of adipocytes [6].

Deficiencies in the formation of lipid droplets are usually associated with impaired differentiation signaling in preadipocytes and often with dysregulated expression of PPARG and CEBPA [7]. Activation of these transcription factors is also responsible for the metabolic switch from glycolysis to mitochondrial oxidative phosphorylation for ATP production during adipocyte differentiation [8] as well as for mediating the expression of lipogenic genes such as perilipin 1 (PLIN1) [9] or fatty acid-binding protein 4 (FABP4) [10].

In contrast to obesity-mediated effects on adipose tissue, escalated inflammatory signaling in remodeling wound tissue exerts a much stronger cellular response that greatly impacts on cell physiology and differentiation properties. Exposure to inflammatory cytokines affects the activation of the differentiation signaling cascade and—depending on the abundance of anti-adipogenic cytokines—induces a metabolic shift towards glycolysis [11].

Although current literature describes some aspects of single cytokine effects on differentiating ASC [12, 13], in-depth analyses of the affected pathways and adaptive ASC response strategies to an inflammatory environment are lacking. Furthermore, characterization of how adipogenesis occurs and how adipocytes behave outside of classical adipose tissue depots is scarce. Herein, we describe the presence of adipocytes in human wound tissue and use validated human macrophage polarization models to characterize the molecular mechanisms of their development throughout the phases of wound healing.

## Material and methods

### Granulation tissue harvesting and preparation

Granulation tissue was harvested from 13 patients undergoing surgical intervention for chronic wounds at our department. The study was approved by the Ethics Committee of the Medical University of Innsbruck (EK0244/2018, EK4368/2016), and written informed consent was obtained from all participating donors. The etiology of wounds included trauma, pressure ulcer, and post-surgical complications. Pictorial representations are from a 53-year male with a chronic wound of the left foot after multiple surgical debridements. Comparative images are of intact fat tissue harvested from a 30-year male who underwent elective abdominoplasty. After harvesting, representative sections of tissue were prepared for immunohistochemistry. Intravital microscopy was performed on a spinning disk confocal system (UltraVIEW VoX; Perkin Elmer, Waltham, MA) connected to a Zeiss AxioObserver Z1 microscope (Zeiss, Oberkochen, Germany) of viable granulation or fat tissue stained with BOPDIPY-493/503 (3 μg/mL, ThermoFischer Scientific, Austria), CF®555 WGA (5 μg/mL, Labconsulting, Austria), and Hoechst 33342 (5 μg/mL, ThermoFischer Scientific, Austria) in phosphate-buffered saline (PBS, PAN Biotech, Germany).

### Immunohistochemistry

Formalin (4%)-fixed and paraffin-embedded human granulation tissue samples were sectioned at 2.5-μM thickness and mounted onto TOMO® Microscope Slides (Matsunami Glass, USA). Deparaffinization and rehydration were performed with xylol and ethanol dilution series. Antigen retrieval was performed in a water bath, by incubation in citrate buffer. The endogenous peroxidase was blocked with 3% H_2_O_2_ prior to antibody incubation. Primary antibody incubation was performed for 24h at 4°C using CD68 (Monoclonal Mouse, Anti-Human CD68, Clone PG-M1, Dako, Denmark) and perilipin (PLIN, Perilipin-1 (D1D8) XP® Rabbit, Cell Signaling Technology, Germany). Specimens were then treated with primary antibody enhancer, horseradish peroxidase polymer, and AEC substrate and then counterstained with hemotoxylin (Scharlau, Spain) and cover-slipped. Images were acquired with a Zeiss AXIO Imager Z2 microscope (Zeiss, Austria).

### Immunofluorescence

For detection of adipocytes and macrophages simultaneously, tissue sections were heat pretreated with citrate buffer and incubated with perilipin1 (PLIN1 (D1D8) XP® Rabbit monoclonal antibodies (mAb), Cell Signaling Technology, Germany) and CD68 (anti-human-CD68 mAb, Clone PG-M1, Dako, Denmark) antibodies. Primary antibodies were visualized with the secondary antibodies donkey-anti-rabbit-Alexa-Fluor-Plus 555 (Invitrogen, California) and donkey-anti-mouse-Alexa-Fluor-Plus 488 (Invitrogen, California). Image acquisition was conducted with Celena S Digital imaging System (Logos Biosystems, South Korea).

### Isolation and culture of human adipose-derived stem cells (ASC) and single-cell suspension of granulation tissue cells

ASC were isolated from subcutaneous abdominal fat tissue obtained from 14 patients (mean (SEM): age 40.6 (15.1), BMI 25.2 (2.7), 10 females, 4 males) undergoing elective abdominoplasty at our department. The study was approved by the Ethics Committee of the Medical University of Innsbruck (EK0244/2018), and written informed consent was obtained from all participating donors. The adipose tissue was washed with PBS (PAN Biotech, Germany), minced into small pieces, and incubated with collagenase type I (0.15% in PBS, Roche, Germany) for 1 h at 37°C. After digestion, samples were centrifuged at 300*g* for 5 min, treated with erythrocyte lysis buffer (BioLegend, Austria) for 5 min, and spun at 300*g* for 5 min. The stromal vascular fraction (SVF) was resuspended in DMEM/F12 medium (PAN Biotech, Germany), filtered through a 100-μm and 40-μm nylon mesh cell strainer (VWR, Austria), counted with a CASY cell counter (Schärfe System, Germany), and plated for culture in PM4 [14] medium consisting of DMEM/F12 medium (PAN Biotech, Germany) supplemented with 1 ng/mL rhFGF2, 10 ng/mL EGF (Immunotools, Germany), 500 ng/mL Insulin (Roche, Austria), 2.5% FCS, and 1% Penicillin/Streptomycin (PAN Biotech, Germany). One hour after plating, nonadherent cells were washed off and attached cells were further cultured inPM4. Adipogenic and osteogenic differentiation potential of ASC was routinely assessed at early passages (passages 2 to 3 after isolation).

Single-cell suspensions from granulation tissue were generated by applying a slightly modified protocol. After extensive washing with PBS, purified granulation tissue specimen were minced into small pieces and incubated with 0.3% collagenase type I until complete digestion (3–5 h). Downstream processing was accomplished according to ASC isolation.

### Generation of macrophage-like conditioned media

For cell expansion, THP1 cells (ATCC TIB202) were cultured in RPMI 1640 medium supplemented with 10% FCS and 1% Penicillin/Streptomycin and 2 mM glutamax (ThermoFisherScientific, Germany). To minimize effects on ASC by distinct culture media, THP1-derived macrophage maturation was performed in DMEM/F12 medium supplemented with 2.5% FCS and was induced by the addition of 25 ng/mL phorbol-12-myristate-13-acetate (PMA dissolved in DMSO) for 24h. The attached cells were washed with PBS, allowed to recover in DMEM/F12 medium supplemented with 2.5% FCS for additional 24 h. Thereafter, cells were activated for 24 h by adding recombinant human IFNG (10 ng/ml) and *Escherichia coli* LPS (10 ng/mL) to generate pro-inflammatory macrophages (M^(IFNG/LPS)^), or addition of recombinant human IL4 (10 ng/mL) and IL13 (10 ng/mL) to generate anti-inflammatory macrophages (M^(IL4/IL13)^). PMA-treated THP1 cells cultured in DMEM/F12/2.5% FCS without stimuli were considered as unstimulated macrophages (MΦ) and untreated THP1 cells maintained in culture without any stimulation as monocytes (Mo). After stimulation, cells were washed three times with PBS to remove the activator cytokines and cultured in DMEM/F12/2.5% FCS for an additional 24 h to generate conditioned media (CM). Finally, CM was harvested, centrifuged for 10 min at 300*g*, and filtered (pore size < 0.2μm) to eliminate any residual cellular components. N-chlorotaurine (NCT) was synthesized as previously described [15]. Solid NCT was resolved in a.bd. and added during the macrophage activation phase at a concentration of 500 μM. All cytokines and LPS were obtained from Peoprotech, Germany.

### Monocyte isolation and macrophage differentiation

CD14 BD IMAG Beads (Becton-Dickinson, BD Austria) were used to isolate monocytes from the blood of normal healthy donors according to the manufacturer’s instructions. Written informed consent was obtained from all participating blood donors by the Central Institute for Blood Transfusion & Immunological Department, Innsbruck, Austria. The use of anonymized leftover specimens for scientific purposes was approved by the Ethics Committee of the Medical University of Innsbruck (EK1166/2018). Monocytes were re-suspended in RPMI medium (PAN Biotech, Germany) containing 5% AB serum and 2 mM l-glutamine and cultured with GMCSF (50 ng/mL) or MCSF (50 ng/mL) for 7 days at 37°C/5% CO_2_. GMCSF-stimulated macrophages were further challenged with recombinant human IFNG (10ng/ml) and *E. coli* LPS (10 ng/mL) for 24h to obtain pro-inflammatory GMCSF^(IFNG/LPS)^ macrophages. MCSF-stimulated macrophages were challenged with recombinant human IL4 (10 ng/mL) and IL13 (10 ng/mL) to obtain anti-inflammatory MCSF^(IL4/IL13)^ macrophages. After stimulation, cells were washed with PBS three times to remove the activator cytokines and cultured in RPMI/2.5% FCS for an additional 24 h to generate conditioned media (CM). Finally, CM was harvested, centrifuged for 10 min at 300*g*, and filtered (pore size < 0.2μm) to eliminate any residual cellular components.

### Antibody array

Macrophage-CM was loaded onto RayBio® Human Cytokine G5 Antibody Microarray Glass Chip (RayBiotech, Norcross, GA) which facilitates the detection of 80 targets. The arrays were processed according to the manufacturer’s instructions. The relative fluorescent intensities of the spots were analyzed using the GenePixx 4000B microarray scanner (Molecular Devices, USA), and specific signal intensities at 532 nm were normalized to background. Conditioned media were examined in duplicates from 3 independent experiments.

### Adipogenic differentiation of human ASC

Adipogenic differentiation was induced using adipogenic induction medium (AIM) based on DMEM/F12 medium supplemented with 2.5%FCS, 1 μM troglitazone, 500 μM 3-isobutyl-1-methyl-xanthine (IBMX), 250 nM T3, 100nM dexamethasone, and 1 μM insulin. After 7 days, AIM was exchanged for adipocyte differentiation medium (ADM) containing 2.5% FCS, 1 μM insulin, and 1 μM troglitazone, and cells were cultured until day 14. All reagents and dyes were obtained from Sigma Aldrich, Germany. For IL1B-inhibition experiments, AIM and ADM were supplemented with 100 ng/mL human recombinant interleukin 1 receptor antagonist (IL1RA, Peprotech, Austria). For microscopy assessment of lipid content, cells were stained with BODIPY-493/503 (1 μg/mL, Sigma Aldrich, Austria) and Hoechst 33342 (1 μg/mL, Sigma Aldrich, Austria) dilution in PBS for 15 min, then washed with PBS, and imaged using Celena S Digital Imaging System. Cell numbers of day 14 differentiated adipocytes were determined by counting Hoechst 33342-positive nuclei per visual field using ImageJ version 1.49m. Three random positions per well were analyzed. For flow cytometry analysis, the same cells were trypsinized, and acquisition performed using a BD-FACS Calibur flow cytometer. Analysis was completed using FlowJo Software version 10.1r1.

### Immunoblotting

5×10^4^ to 2×10^5^ cells were directly lysed in Laemmli buffer containing 10% dithiothreidol (DTT, Sigma Aldrich, Germany), sonicated, and boiled for 5min at 75°C. Proteins were size fractioned on pre-stained gradient polyacrylamide gels (MiniPROTEAN®TGX Stain-Free™ Precast Gels, Biorad, Germany), blotted onto 0.2μm PVDF membrane, blocked for 2 h in 5% reconstituted low fat milk powder. Membranes were incubated overnight with primary antibodies against HK1, HK2, PFKP, PKM2, LDHA, FASN, ACC, ACSS2, ACLY, PLIN1, FABP4, CEBPA, CEBPB, PPARG, and GAPDH (all obtained from Cell Signaling, Germany) in Tween-TBS (TTBS) supplemented with 5% BSA. SREBP1 (clone 2A4) antibody was obtained from Novus Biological. After extensive washing, horseradish peroxidase-conjugated sheep-anti-mouse and sheep-anti-rabbit antibodies (Cell Signaling, Germany) were incubated for 1 h, and the reaction was visualized by enhanced chemiluminescence reagent ECL (BioRad, Germany) using a BioRad ChemidocMP gel analyzer for detection. Quantification was carried out using the ImageLab 5.0 software (Biorad, Germany) according to the manufacturer’s instructions, with normalization of protein expression to total protein loading.

### Plasmid construction and lentiviral transduction

For constitutive PPARG overexpression, human PPARG-cDNA (pDONOR223_PPARG_WT [16]) was cloned by LR-recombination according to the manufacturer’s instruction into the lentiviral transduction plasmid pHR-SFFV-DEST-IRES-PURO [17], thereby generating pHR-SFFV-PPARG^WT^-IRES-PURO. Generation of pHR-SFFV-PURO plasmid has been described previously [18]. For generation of lentiviral particles, 1.5 μg of sequenced verified plasmids was co-transfected with 0.9 μg pSPAX2 packaging and 0.9 μg pMD-G (VSV-G-) pseudotyping plasmids using calcium phosphate transfection. Supernatants were harvested 48 h and 72 h after transfection, filtered (pore size < 0.2μm), and diluted 1:2 with fresh PM4 medium supplemented with 1 μg/mL polybrene for infection. ASC were selected for puromycin resistance (1 μg/mL) 48 h after infection. All reagents were obtained from Sigma Aldrich, Germany.

### RNA isolation and quantitative RT-PCR

RNA was isolated using Trizol-Reagent (Sigma Aldrich, Germany), and cDNA was synthesized using random hexamer primers and iScript cDNA-synthesis kit (BioRad, Germany) as previously described [19]. The RT-qPCR reactions were performed using the SsoAdvanced™ Universal SYBR® Green Supermix kit (Biorad, Germany) on a CFX96-RT-qPCR machine (BioRad, Germany) using following protocol: 95°C for 2 min, 40 cycles of 95°C (15s), 60°C (15s), and 72°C (10s). Gene expression was determined by using the BioRad CFX Manager 3.1 software, Ct values were normalized to the mean expression of the reference gene 18S rRNA and are presented as –ΔCt. Oligonucleotides used for gene expression assays are listed in Supplement Table 3. Referenced and newly designed primers used in this study were synthesized by Microsynth Austria, and specificity was tested by assessment of the melting curve.

### Statistics

All experiments were repeated independently at least three times using cells from different donors. To assess the quality of data, descriptive statistics were performed, using Kolmogorov Smirnov testing for Gaussian distribution of the values. Significances were tested by Student’s *t* test, Mann-Whitney, and one-way ANOVA test in combination with Dunnett’s test for multiple comparison. Data are presented as mean ± SEM. *P* values of <0.05 were considered statistically significant. Statistical analyses were carried out using Prism 7 (Graphpad software Inc., version 5.0). Heatmap analysis was performed in R (https://r-project.org) version 3.4.3.

## Results

### Adipocytes are present in human granulation tissue

**Fig. 1:**
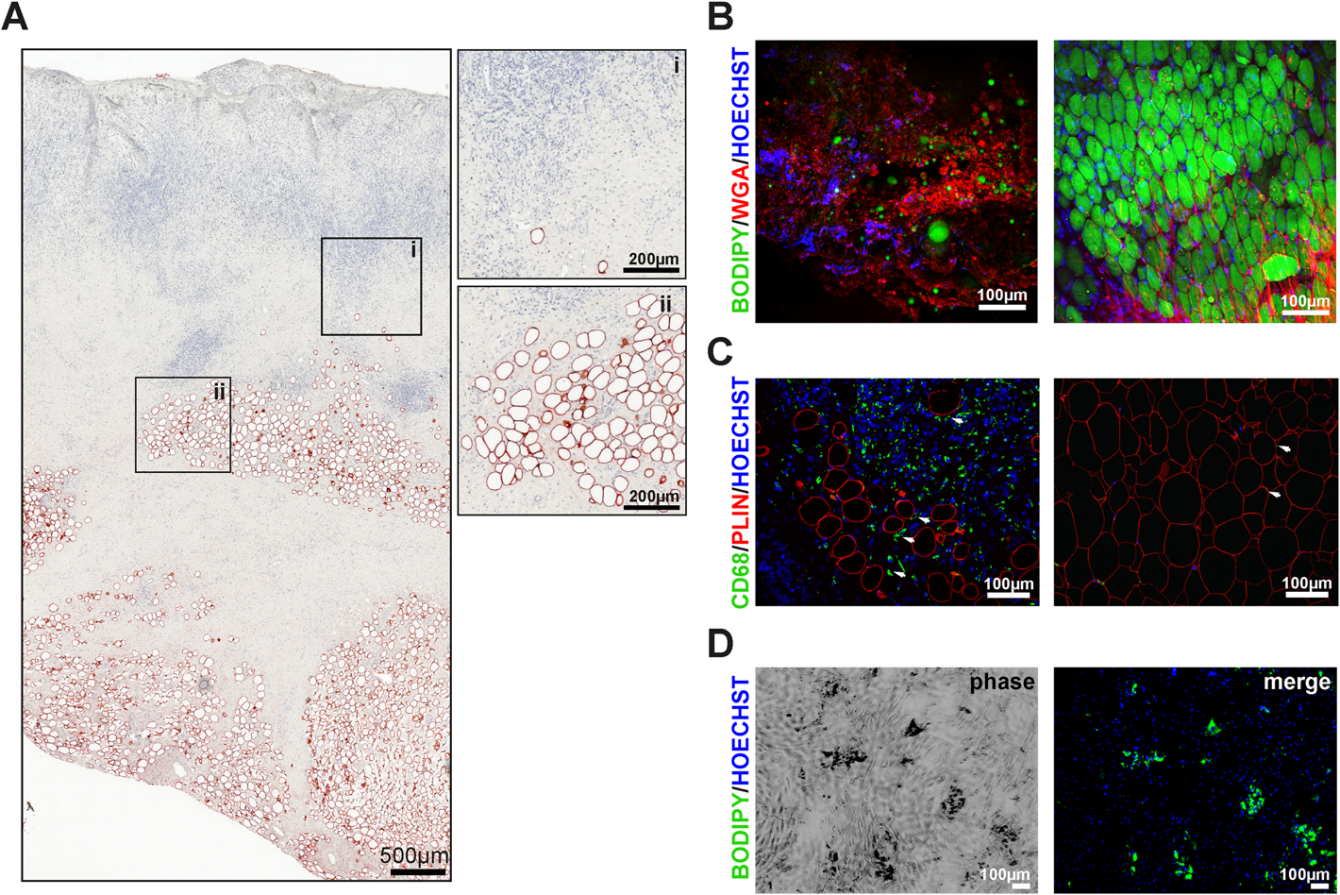
Adipocytes are present in human granulation tissue of chronic wounds. **a** Histology of a representative granulation tissue section isolated from a 51-year-old patient (male) with a chronic lower limb wound and stained with hematoxylin and eosin (H&E) and PLIN1 (red staining). Areas highlighted are at higher magnification and indicate the presence of single (i) and clustered adipocytes (ii). **b** Intra-vital confocal microscopy of unfixed granulation tissue (same patient, left panel) and intact fat tissue (30-year-old male patient, right panel) stained with neutral lipid sensitive dye BODIPY493/503 (green), CF®555-labbeled wheat germ agglutinin to highlight *N*-acetyl-d-glucosamine and sialic acid present in heterogeneous polysaccharides (e.g., hyaluronan) of extracellular matrices (ECM), and Hoechst 33342 staining for nuclei (blue). **c** Double staining of representative sections from granulation tissue (left panel) or intact fat tissue (right panel) with CD68 (AF488, green) and PLIN (AF555, red). Nuclei (blue) were counterstained with Hoechst 33342. The arrows heads indicate CD68 positive cells (macrophages) in close proximity to adipocytes. **d** Single-cell suspension from granulation tissue was subjected to in vitro differentiation for 14 days. Lipid droplets and nuclei were visualized by BODIPY493/503 (green) and Hoechst 33342 (blue), respectively

Sections of granulation tissue were isolated from patients with chronic non-healing wounds requiring surgical debridement. Representative samples were fixed for histological examination and stained for adipocyte maker PLIN1, a protein associated with lipid droplets. We noted the consistent appearance of PLIN1-positive cells within the heterogeneous architecture of granulation tissue across multiple patient samples (Fig. 1). Of note, single adipocytes were present in the superficial layer of granulation tissue and showed a more clustered organization as tissue depth progressed (Fig. 1a). These adipocytes were clearly separated from subcutaneous fat tissue by connective tissue and embedded in a hyaluronan and polysaccharide-enriched extracellular matrix (ECM) as visualized with fluorescence labeled wheat germ agglutinin WGA555 (Fig. 1b). Moreover, single and clustered adipocytes reside in areas with increased numbers of CD68^+^ macrophages, which seemed not to be detrimental for adipocyte differentiation (Fig. 1c). However, the discernible, inconsistent pattern of adipocytes within granulation tissue of distinct patient specimens (Table 1) suggested the existence of specific areas, which support or dampen adipocyte differentiation by yet unknown mechanisms. To prove that cells from granulation tissue are capable of differentiating into adipocytes, single-cell suspensions from selected granulation tissue specimens were exposed to an adipogenic differentiation cocktail in vitro and assessed for the formation of lipid droplets (Fig. 1d). Although the frequency was low, granulation tissue resident cells differentiated into adipocytes. Since macrophages are a predominant cell type within granulation tissue [20], we hypothesized that the specific distribution of adipocytes might be defined by clusters of polarized macrophages, which are known to exert pro- and anti-adipogenic effects in classical adipose depots.

**Table 1:** Characteristics of patients included for the analysis of granulation tissue

Patient	Age (years)	Gender	Wound type	Location	Wound condition (days)	Adipocytes (PLIN1 pos. cells)
1	50-59	2	Infection	Foot	18	+++
2	80-89	1	Pressure Ulcer	Sacrum	12	+++
3	50-59	2	Trauma	Foot	13	+++
4	60-69	1	Post-Operative - Malignancy	Thigh	97	++
5	70-79	2	Infection	Knee	11	-
6	90-100	2	Pressure Ulcer	Sacrum	7	++
7	40-59	1	Pressure Ulcer	Hip	13	+
8	40-49	2	Pressure Ulcer	Hip	57	-
9	18-27	2	Pressure Ulcer	Hip	n.a	+
10	50-59	2	Pressure Ulcer	Hip	14	+
11	40-49	1	Pressure Ulcer	Hip	22	++
12	50-59	2	Pressure Ulcer	Hip	14	+
13	70-79	1	Infection	Breast	14	++

Granulation tissue patient characteristics, including wound localization, etiology (wound type), days of wound condition (after first debridement until granulation tissue harvest), and estimated frequency of adipocytes (+ indicates single adipocytes, ++ indicates for single adipocytes and small adipocyte cluster, +++ single adipocytes, and many adipocyte cluster—no adipocytes detected)

### Mimicking distinct macrophage polarization in a human in vitro model

**Fig. 2:**
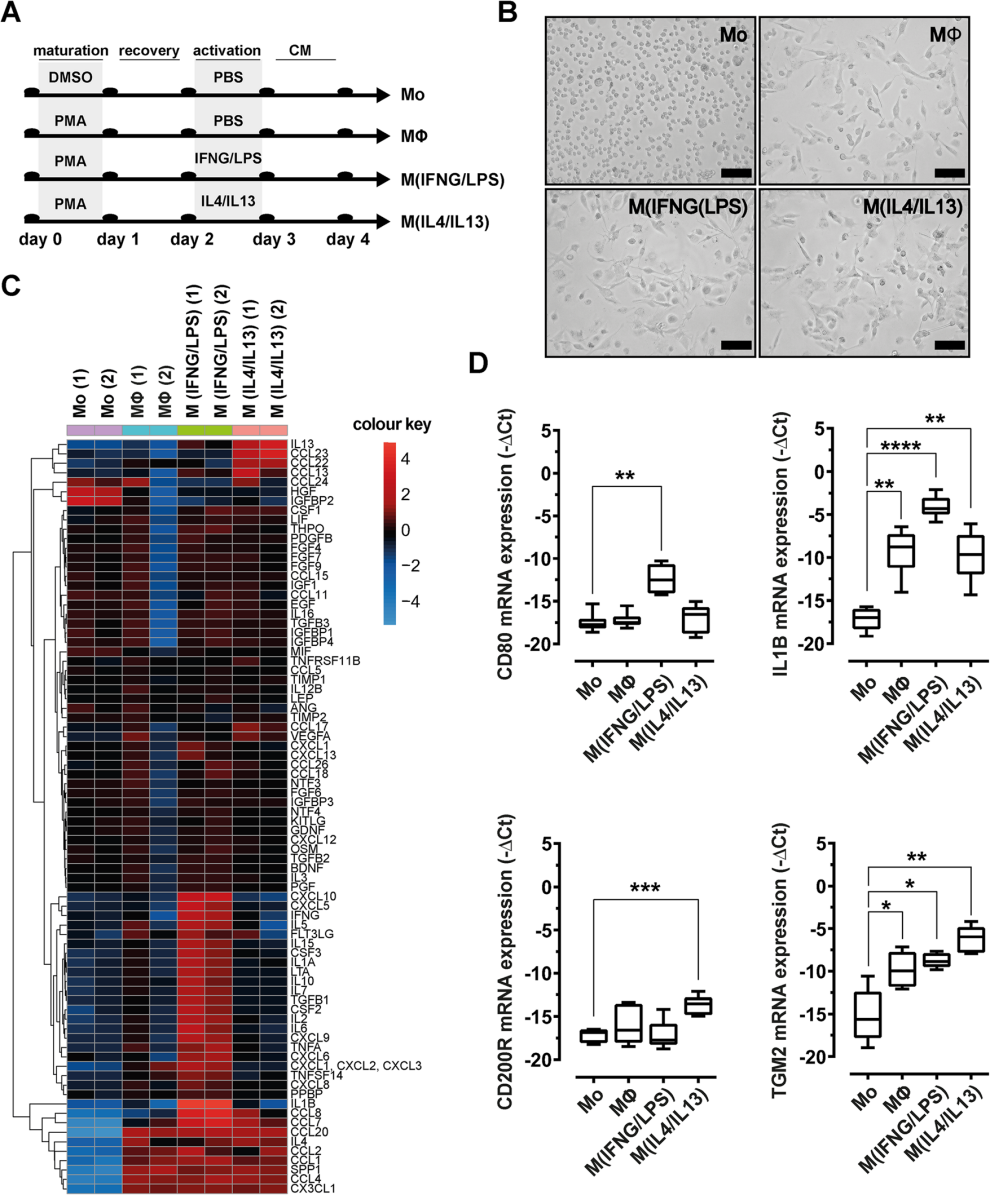
Characterization of the macrophage activation model. **a** THP1 cell treatment protocol for in vitro polarization of macrophages. **b** Morphology (phase contrast, scale bar 100μm) of THP1-derived macrophages on day of CM harvest. **c** Heatmap of log2 cytokine protein levels in differentially activated macrophages. The dendrogram represents the result from a complete linkage hierarchical clustering of mean-centered expression levels based on Euclidian similarity. **d** mRNA-expression levels of macrophage-selective markers (M^(IFNG/LPS)^: CD80, IL1B, and M^(IL4/IL13)^: CD200R, TGM2). Data were normalized to the 18S rRNA reference gene and shown as mean –ΔCt. Asterisks indicate *p* values < 0.05 (*), *p*<0.01 (**), and *p*<0.001 (***). Statistical significance (*n*=5) was determined by using one-way ANOVA and Dunnett’s test for multiple comparison

To test our hypothesis that macrophages were the regulating factor for pro- and anti-adipogenic signaling in granulation tissue, we established an in vitro macrophage polarization model based on the human monocytic cell line THP1 (Fig. 2a). We adapted the macrophage model to enable paired analyses with human ASC, by accustoming THP1 cells to ASC basic DMEM/F12 medium supplemented with low FCS concentration (2.5%). The model was further optimized for the concentrations of phorbol-12-myristate 13-acetate (PMA), recovery time, and stimulatory cytokines, to induce polarization towards inflammatory macrophages stimulated by IFNG/LPS (M^(IFNG/LPS)^), or anti-inflammatory macrophages stimulated by IL4/IL13 (M^(IL4/IL13)^). PMA treatment induced cell adherence and marked changes in cell shape that was further altered by the addition of activating cytokines (Fig. 2b). Differences in the macrophage secretome were qualitatively determined by profiling of 84 cytokines using antibody array and RT-qPCR for selected marker gene expression. Clustered heat map analyses of obtained cytokine profiles suggested pronounced differences in the secretory profile of THP1 (Mo) and THP1-PMA (MΦ, M^(IFNG/LPS)^, M^(IL4/IL13)^)-treated cells (Fig. 2c, Supplement Table 1). PMA-treated cells resembling an unstimulated macrophage phenotype were characterized by high levels of CCL20, CCL2, SPP1, and CCL4 and low levels HGF and IGFBP2, respectively. Upon stimulation with IFNG/LPS, classical pro-inflammatory cytokines were induced including IL1B, IFNG, and TNFA. Additional cytokines such as IL2, IL6, IL8, and CXCL10, known to be associated with inflammatory signaling and impaired wound healing, were induced in M^(IFNG/LPS)^ cells. In contrast, M^(IL4/IL13)^ macrophages showed increased levels of anti-inflammatory cytokines such as IL13 and CCL23. Unstimulated monocyte cells (Mo) showed high levels of HGF and IGFBP1, but were negative for the inflammatory cytokines IL1B, CXCL10, and CCL2. The macrophage polarization model was further validated by quantitative RT-qPCR analysis of additional surface markers and selected cytokines (Fig. 2d). These analyses revealed that M^(IFNG/LPS)^ macrophages expressed significantly higher levels of CD80 and IL1B than M^(IL4/IL13)^ macrophages, which instead showed a higher expression of CD200R and transglutaminase 2 (TGM2).

### M^(IFNG/LPS)^-conditioned medium attenuates adipogenesis

**Fig. 3:**
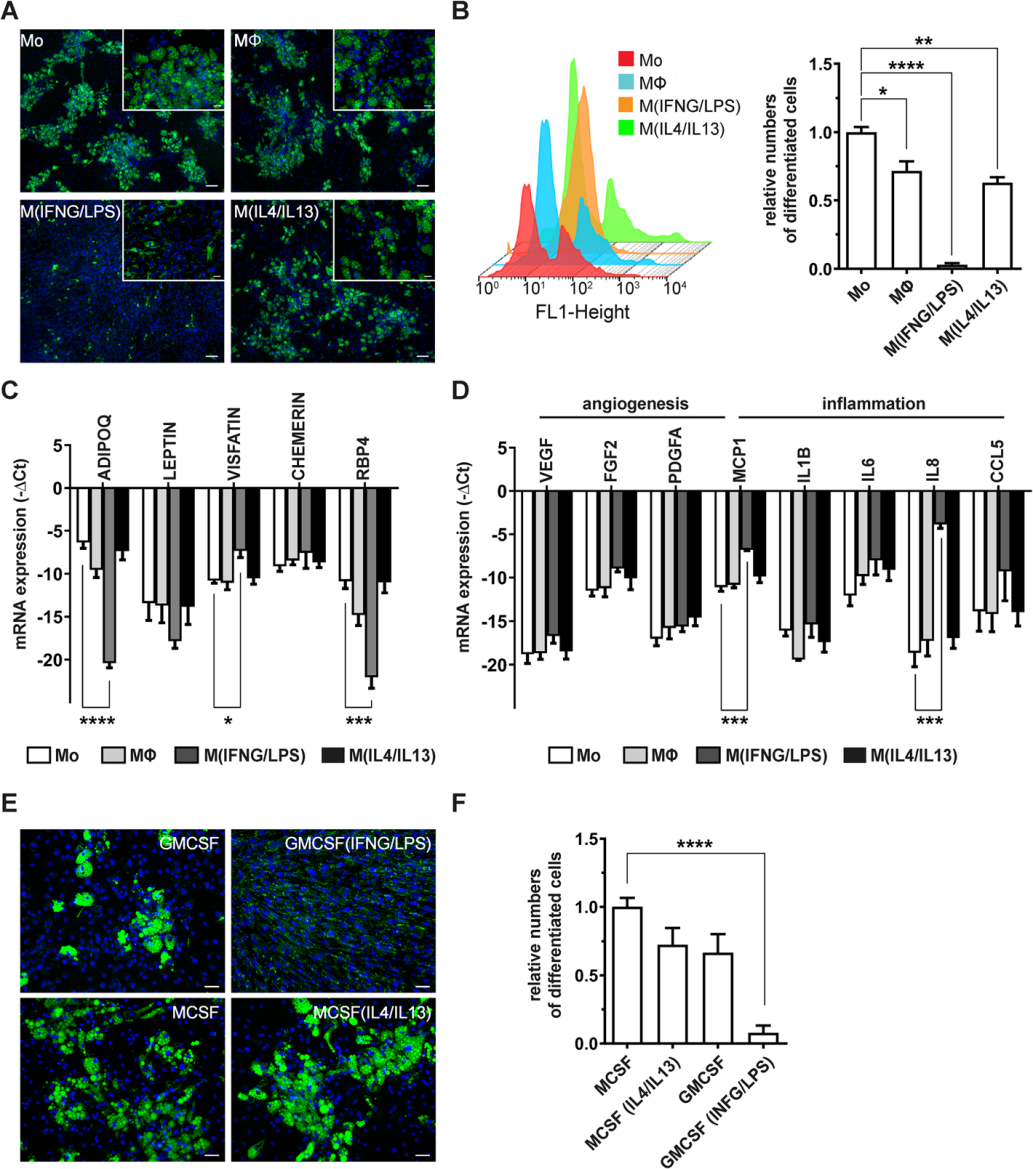
M^(IFNG/LPS)^ CM impairs the formation of mature lipid-laden adipocytes. **a** Representative images of human ASC differentiated to adipocytes for 14 days in the presence of distinct macrophage-CM. Lipid droplets and nuclei were visualized by BODIPY493/503 (green) and Hoechst 33342 (blue), respectively. Sizebar = 100μm. **b** Flow cytometry analysis of differentiated adipocytes at day 14. Shown are an overlay of histrograms (left panel) and relative numbers of large lipid containing adipocytes (stained with BODIPY-493/503) compared to Mo-CM-treated cells, *n*=6. **c**, **d** mRNA-expression levels of selected adipokines (**c**) and angiogenesis- or inflammation associated cytokines (**d**) in day 14 differentiated adipocytes. Data were normalized to the 18S rRNA reference gene and shown as mean –ΔCt, *n*=3. **e** Representative microscopy images of day 14 differentiated adipocytes exposed to CM of pro- and anti-inflammatory CD14^+^ macrophage-CM. **f** Quantification of large lipid droplet containing cell numbers exposed to CD14^+^ macrophage-CM after 14 days of differentiation, *n*=4. Asterisks indicate *p* values <0.05 (*), <0.01 (**), <0.001 (***), and <0.0001 (****). Statistical significance was determined by using one-way ANOVA and Dunnett’s test for multiple comparison. Data are shown as mean ± SEM

Next we applied the macrophage polarization model to primary human ASC during differentiation into adipocytes. As shown in Fig. 3a, conditioned medium (CM) harvested from activated macrophages induced distinct ASC differentiation phenotypes. CM of unstimulated- (MΦ) or IL4/IL13-stimulated macrophages (M^(IL4/IL13)^) exhibited moderate anti-adipogenic effects compared to ASC exposed to CM from monocytic cells (Mo). In sharp contrast, M^(IFNG/LPS)^-CM strongly induced aberrant accumulation of lipid droplets and almost completely abolished formation of large lipid vacuoles in day 14 differentiated ASC. These cells were characterized by numerous tiny little lipid droplets (Fig. 3a, magnification), which only became visible after staining with BODIPY 493/503, a dye staining neutral lipids such as triacylglycerols (TAG) or cholesterol in cells. Using flow cytometry-based analysis of differentiated cells, M^(IFNG/LPS)^-CM differentiated cells shifted as a single population in the FL1-H channel, whereas treatment with all other CM showed two clearly separated populations (Fig. 3b, left panel). To avoid biased quantification of differentiated adipocytes by the M^(IFNG/LPS)^-CM induced phenotype in our flow cytometry analysis, we therefore considered only cells containing large lipid droplets as differentiated adipocytes in our analysis (Fig. 3b, right panel). Applying this analysis strategy, M^(IFNG/LPS)^-CM reduced adipocyte differentiation by more than 95% (numbers of large lipid droplet containing cells (mean (SEM) = 4.1% (2.3)) compared to Mo-CM differentiated cells. In contrast, MΦ-CM (mean (SEM) = 71.6% (6.3)) and M^(IL4/IL13)^ CM-treated ASC (mean (SEM) = 63.5% (4.8)) moderately reduced numbers of differentiated adipocytes. Of interest, the M^(IFNG/LPS)^-CM mediated effect on differentiating adipocytes was not limited to lipid droplet formation, but was also apparent on cytokine production in these cells (Fig. 3c, d). M^(IFNG/LPS)^-CM differentiated cells expressed low levels of the adipokines adiponectin (ADIPOQ) and retinol binding protein 4 (RBP4) but high levels of inflammatory cytokines such as MCP1 and IL8. Thus, exposure to CM harvested from macrophages stimulated with IFNG/LPS, but not IL4/IL13, impacted lipid droplet formation in human ASC and induced a pro-inflammatory cytokine pattern in these cells.

### IFNG/LPS stimulated human-derived CD14^+^ monocytes confirm macrophage effects on ASC differentiation

We confirmed the anti-adipogenic effect of IFNG/LPS stimulated macrophages with monocytes isolated from human peripheral blood samples of healthy donors. In line with our THP1-based macrophage model, CD14^+^ monocytes enriched from PBMC were cultured in the presence of GMCSF or MCSF and stimulated for 24h with IFNG/LPS to generate pro-inflammatory macrophages (GMCSF^(IFGN/LPS)^), or IL4/IL13 to generate anti-inflammatory macrophages (MCSF^(IL4/IL13)^), respectively. Akin to our THP1-model, pro-inflammatory GMCSF^(IFNG/LPS)^—stimulated macrophages inhibited development of large lipid droplets as observed by M^(IFNG/LPS)^ macrophages, whereas MCSF^(IL4/IL13)^ stimulated macrophages had minor effects on the formation of large lipid droplets (Fig. 3e, f).

### M^(IFNG/LPS)^ macrophages impact glycolysis and lipid metabolism in differentiating ASC

**Fig. 4:**
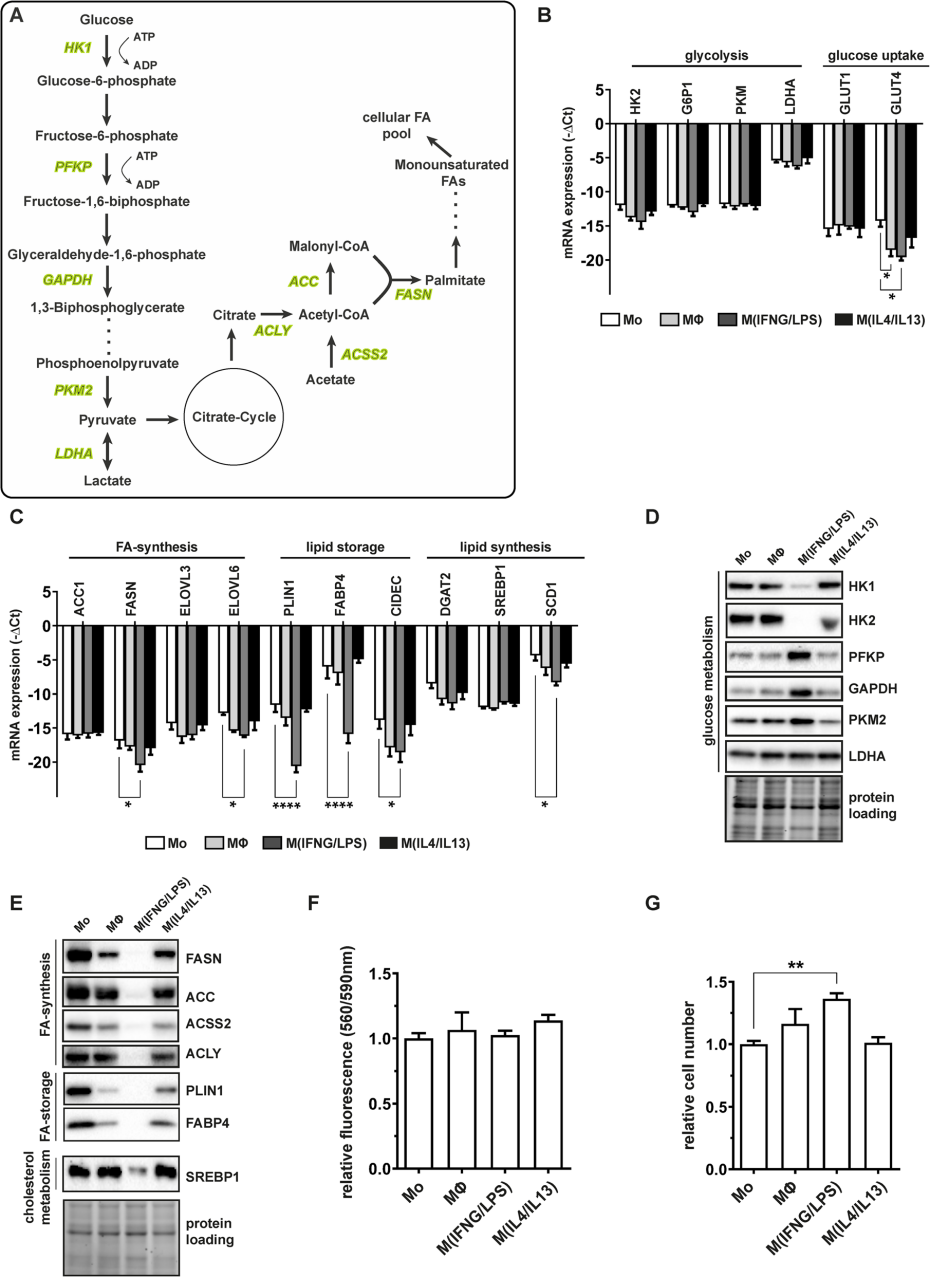
M^(IFNG/LPS)^ macrophage impair endogenous fatty acid synthesis in ASC. **a** Simplified overview of the glycolytic and fatty acid synthesis pathways with enzymes analyzed on protein level (marked in green). **b**, **c** mRNA levels of glycolysis - (**b**) and endogenous FA-synthesis regulating (**c**) enzymes analyzed in day 14 CM-differentiated adipocytes. Data were normalized to the 18S rRNA reference gene and shown as mean –ΔCt, *n*=3. **d**, **e** Representative immunoblots of enzymes regulating glycolysis (**d**) or lipid metabolism (**e**) of day 14 differentiated adipocytes. **f** Mitochondrial activity of day 14 differentiated adipocytes assessed by resazurin-based fluorescence assay (PrestoBlue^TM^), *n*=4. **g** Cell counts of day 14 differentiated adipocytes shown as relative numbers compared to Mo-CM-treated cells, *n*=4. Asterisks indicate *p* values <0.05 (*), <0.01 (**), <0.001 (***), and <0.0001 (****). Statistical significance was determined by using one-way ANOVA and Dunnett’s test for multiple comparison. Data are shown as mean ± SEM

To unravel possible molecular mechanisms responsible for abnormal lipid accumulation, we systematically investigated expression of key enzymes regulating glucose metabolism and endogenous fatty acid (FA) synthesis (Fig. 4a). With the exception of glucose uptake transporter 4 (GLUT4), which was downregulated in M^(IFNG/LPS)^ CM-treated cells, mRNA levels of all other investigated enzymes were not susceptible to pro-inflammatory macrophage cytokines (Fig. 4b). In contrast, at protein level, a specific regulation of glycolytic enzymes by M^(IFN/LPS)^-CM was observed. As shown in Fig. 4d, levels of both hexokinases 1 and 2 (HK1 and HK2), which catalyze the first step in glycolysis, declined upon exposure to M^(IFN/LPS)^-CM. However, levels of the downstream glycolytic enzymes, i.e., phosphofructokinase (PFKP), glyceraldehyde-3-phosphate dehydrogenase (GAPDH), pyruvate kinase 2 (PKM2), and lactate dehydrogenase (LDHA), which is activated by hypoxia, significantly increased.

Next we examined whether the key enzymes regulating endogenous FA synthesis are affected by the distinct macrophage-CM. RT-qPCR analysis of enzymes involved in FA- or lipid-synthesis revealed moderate downregulation upon exposure to M^(IFNG/LPS)^-CM, including fatty acid elongases (ELOVL3 and ELOVL6), sterol regulatory element-binding protein (SREBP1; cholesterol synthesis), diacylglycerol O-acyltransferase 2 (DGAT2; triacylglycerol synthesis), and SREBP1 target gene stearoyl-CoA-desaturase 1 (SCD1). More impressively, mRNA levels of proteins involved in the maturation of lipid droplets and intracellular transport of fatty acids such as cell death inducing DFFA like effector c (CIDEC), PLIN1, or FABP4, showed a clear decline on mRNA level (Fig. 4c). The extent of regulation was also confirmed on protein level. Interestingly, protein levels of the acteyl-CoA carboxylase (ACC), which catalyzes irreversible carboxylation of acetyl-CoA to malonyl-CoA, and fatty acid synthetase (FASN), which initiates the first steps of long chain fatty acid synthesis, were more strongly reduced in M^(IFNG/LPS)^ CM-treated cells than estimated from mRNA results (Fig. 4e). Levels of other enzymes regulating endogenous lipid metabolism such as acetyl-CoA synthetase 2 (ACSS2), ATP-citrate lyase (ACLY), or SREBP1 were strongly downregulated by macrophage-CM too, suggesting that endogenous fatty acid metabolism was one of the major targets of macrophage-CM treatment.

Finally, we addressed whether the observed molecular regulations correlated with cell physiological parameters such as mitochondrial respiration and cell proliferation. Neither resazurin-based fluorescence determination of aerobic mitochondrial respiration (Fig. 4f) nor cell count of day 14 differentiated cells (Fig. 4g) revealed major differences between the individual macrophage-CM. Rather, we observed an increase of these physiological markers, further supporting our previous findings that impaired fatty metabolism is causative for the M^(IFNG/LPS)-^CM induced anti-adipogenic effects.

### M^(IFNG/LPS)^-CM impacts on CEBPB levels and impairs PPARG induction

**Fig. 5:**
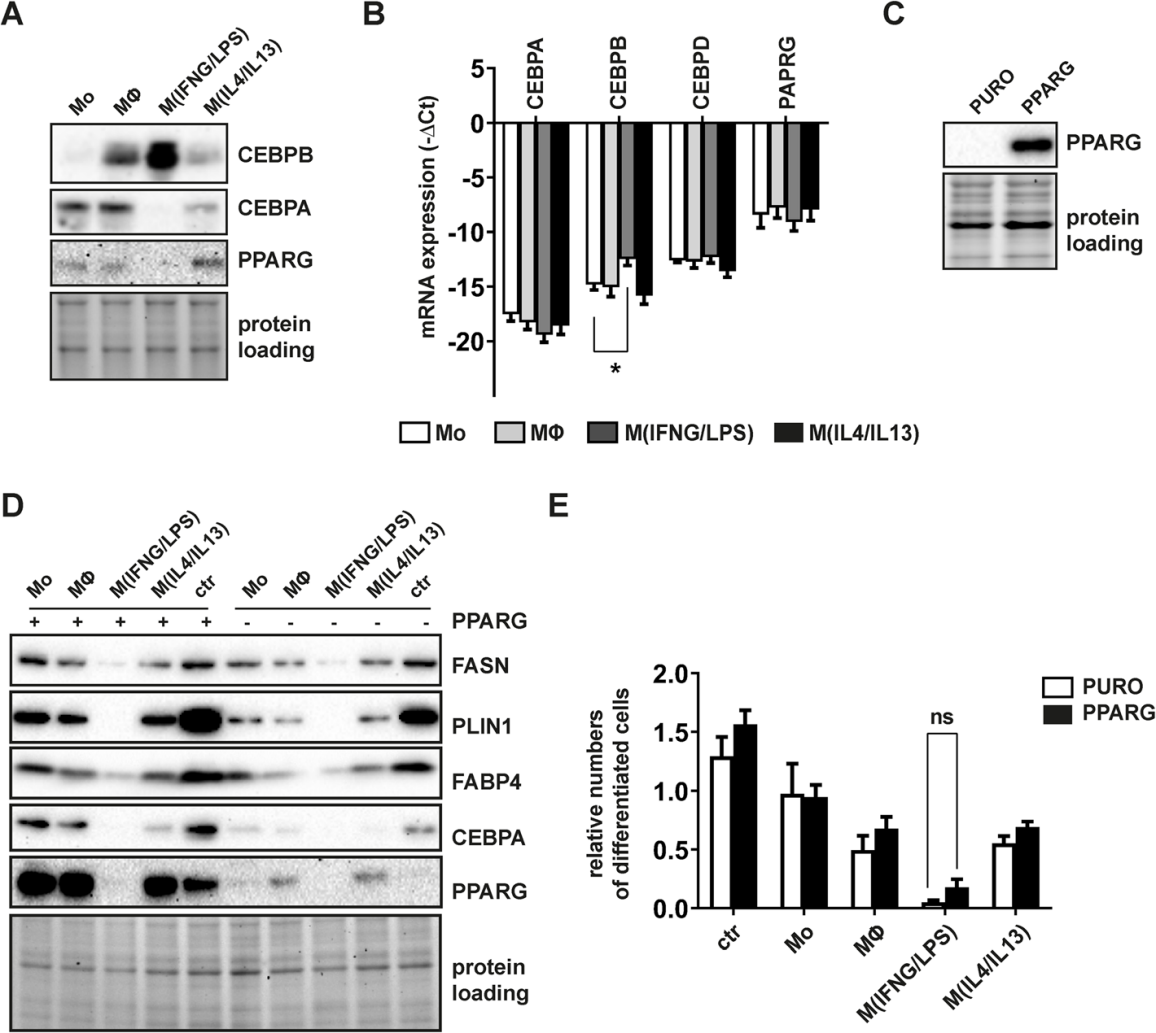
PPARG overexpression was insufficient to rescue anti-adipogenic effects of M^(IFNG/LPS)^ macrophages. **a** Representative immunoblots of the adipogenesis transcription factors CEBPB, PPARG, and CEBPA in early differentiating ASC (day 3) exposed to macrophage-CM. **b** Corresponding mRNA levels of day 3 differentiated cells. Data were normalized to the 18S rRNA reference gene and shown as mean –ΔCt, *n*=3. **c** Representative immunoblot of PPARG in proliferating ASC 72h after lentiviral transduction. **d** Immunoblotting of adipocyte marker proteins in PPARG- and puromycin resistance gene transduced ASC differentiated to adipocytes for 14 days in the presence of macrophage-CM. **e** Flow cytometry analysis of day 14 differentiated PPARG- or puromycin resistance overexpressing ASC. Values depict relative numbers of large lipid containing adipocytes (stained with BODIPY-493/503) compared to Mo-CM-treated cells, *n*=4. For panel **b**, statistical significance was determined by one-way ANOVA and Dunnett’s test for multiple comparison, for panel **e**, *p* values were calculated by unpaired Student’s *t* test. Data are shown as mean ± SEM

To further elucidate the molecular mechanisms affecting lipid metabolism in M^(IFNG/LPS)^ CM-treated cells, we analyzed the expression of adipogenesis regulators CEBPB, CEBPA, and PPARG in early differentiating cells. While PPARG and CEBPA were downregulated in day 3, differentiated cells exposed to M^(IFNG/LPS)^-CM, levels of CEBPB increased, both on mRNA and protein levels (Fig. 5a, b). To investigate, whether PPARG deficiency in M^(IFNG/LPS)^ CM-treated cells was causative for impaired adipogenesis, we transduced ASC with lentiviral vectors constitutively expressing cDNA encoding sequences for human PPARG^WT^ or puromycin resistance gene (Fig. 5c) and subjected these cells to adipocyte differentiation in the presence of distinct macrophage-CM. Although transduced PPARG was functional, as shown by increased differentiation of control infected ASC (not subjected to any CM) and overall increased levels of differentiation markers, PPARG overexpression was not sufficient to rescue the anti-adipogenic phenotype. Neither the number of lipid-laden cells (Fig. 5d) nor the protein levels of FASN, PLIN1, FABP4, or CEBPA were restored by PPARG-overexpression in M^(IFNG/LPS)^ CM-treated cells (Fig. 5e). Of note, although all differentiating cells were derived from the same transduced cell pool (Fig. 5c) and PPARG expression was comparably high in all day 3 differentiated cells (Supplement Figure 2), transgenic PPARG levels were reduced in day 14 differentiated cells exposed to M^(IFNG/LPS)^-CM. These results suggested that PPARG deficiency and possibly inactive CEBPB signaling were responsible for impaired adipogenesis in M^(IFNG/LPS)^ CM-treated cells.

### N-chlorotaurine treatment of macrophages restores adipogenesis in M^(IFNG/LPS)^ CM-treated ASC

**Fig. 6:**
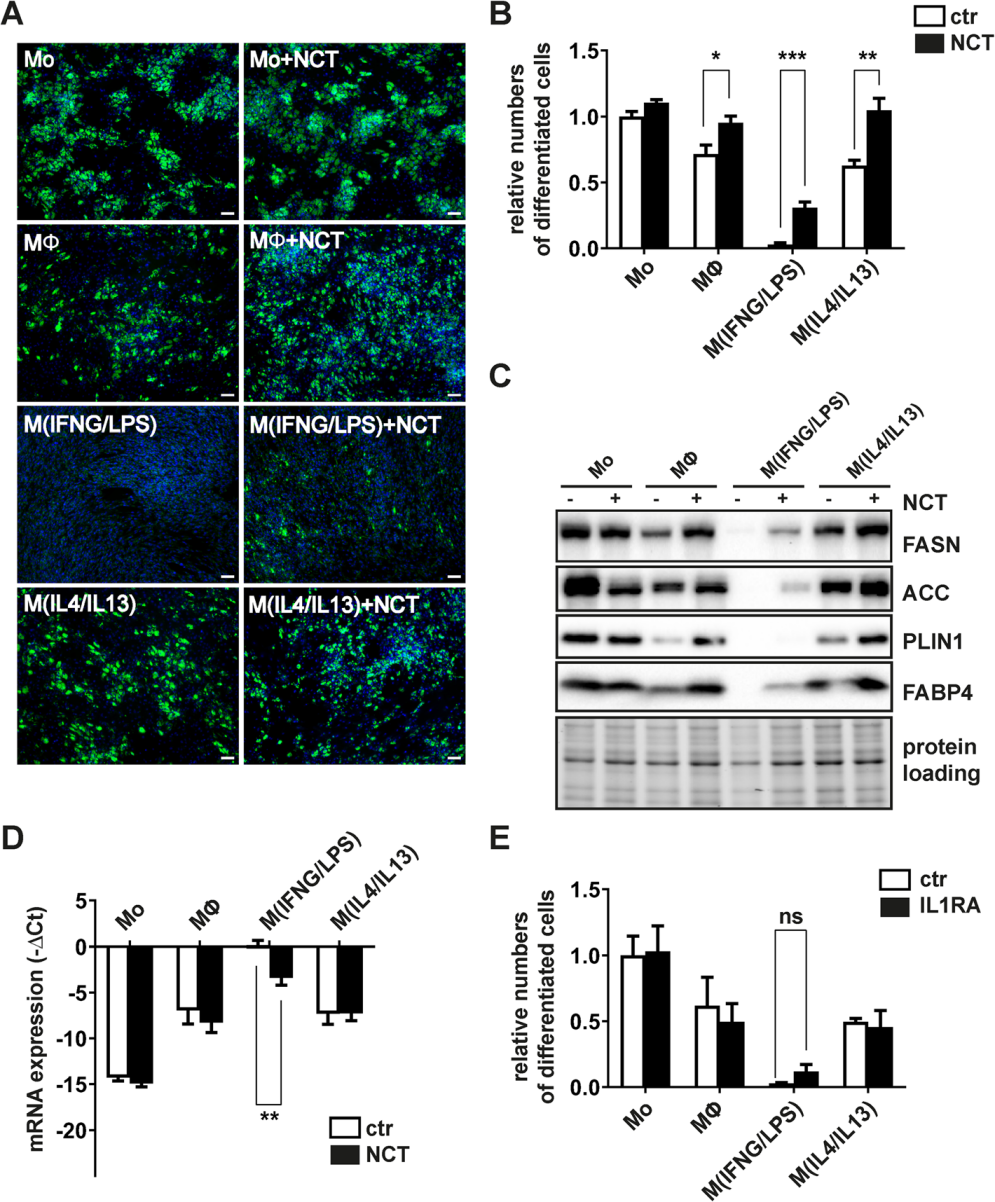
NCT-treatment attenuates the anti-adipogenic effects of M^(IFNG/LPS)^ macrophages. **a** Representative microscopy images of day 14 differentiated adipocytes exposed to macrophage-CM during the differentiation. Macrophages were either co-treated with PBS or NCT (500 μM) during their activation period. **b** Flow cytometry analysis of day 14 differentiated adipocytes cultured in the presence of macrophage-CM during differentiation. Values depict relative numbers of large lipid containing adipocytes compared to Mo-CM-treated cells, *n*=6. **c** Representative immunoblots analyzing expression levels of the adipocyte marker proteins FASN, ACC, PLIN1, and FABP4 in day 14 differentiated cells. **d** IL1B mRNA expression in macrophages co-treated with or without NCT during their activation. Data were normalized to the 18S rRNA reference gene and shown as mean –ΔCt, *n*=4. **e** Flow cytometry analysis of adipocytes differentiated in the presence of macrophage CM for 14 days supplemented with IL1B-inhibitor IL1RA or PBS (ctr). Values depict relative numbers of large lipid containing adipocytes compared to Mo-CM-treated cells, *n*=3. Asterisks indicate *p* values < 0.05 (*), <0.01 (**), and <0.001 (***), respectively. Statistical significance was determined by using non parametric unpaired Student’s *t* test. Data are shown as mean ± SEM

Transgenic PPARG expression in ASC was insufficient to rescue cells from the anti-adipogenic effect mediated by M^(IFNG/LPS)^-CM. Hence, we adapted our strategy and trialed a pharmaceutical compound with clinical relevance to chronic wound healing: N-chlorotaurine (NCT). NCT is an N-chloro derivative of the amino acid taurine, which is produced in humans by activated granulocytes and macrophages and behaves as a mild long-acting oxidant. It can be synthesized chemically as a sodium salt (NCT, Cl-HN-CH_2_-CH_2_-SO_3_^-^Na^+^) [15, 21], which can be used as endogenous antiseptic in aqueous solution with microbicidal activity enhancing chronic wound healing [22, 23]. We co-treated macrophages during their activation phase with non-toxic concentrations of NCT (500 μM) and harvested the CM from macrophages after extensive washing with PBS to remove traces of NCT. As shown in Fig. 6, NCT treatment of macrophages reduced their anti-adipogenic effects, most effectively in IFNG/LPS-activated macrophages (mean (SEM) = 6.4% (5.9) for M^(IFNG/LPS)^ vs. 33.5% (11.6) for M^(IFNG/LPS+NCT)^ (Fig. 6a and b). This effect was also detectable on a molecular level by increased expression of FASN, ACC, PLIN, and FABP4 (Fig. 6c).

The data suggested that NCT mitigates the secretion of adipogenesis-inhibiting cytokines by macrophages. To address this issue, we performed a cytokine array using NCT-treated macrophage phenotypes and compared the cytokine expression levels to control samples. NCT modified the expression of at least 22 cytokines within M^(IFNG/LPS)^-CM suggesting that the observed improvement in adipogenesis is likely to be related to its ability to moderate the pro-inflammatory environment (Supplement Figure 3, Supplement Table 2). In particular, NCT altered the expression of IL1B (Fig. 6d), which is known to inhibit adipogenesis [24, 25] and which was increased in M^(IFNG/LPS)^-CM (Fig. 2d). Previous studies using the IKK2-inhibitor sc-514 suggested that IL1B exerts its anti-adipogenic effect via the NF-kappa B pathway [24]. Therefore, we trialed interleukin receptor antagonist (IL1RA) to competitively block the IL1-receptor from being activated by IL1B. M^(IFNG/LPS)^-CM was supplemented with IL1RA at a concentration abrogating IL1B mediated anti-adipogenic effects of up to 1 ng/mL of recombinant IL1B (Supplement Figure 4). However, IL1B inhibition alone was not sufficient to improve adipogenesis in the presence of M^(IFNG/LPS)^-CM (Fig. 6e).

## Discussion

This study aimed at describing the existence of adipocytes within the human wound healing process and to understand the cellular milieu which stimulates their presence and impacts their function. Together with macrophages, adipocytes have been shown to participate in the clearance of cellular debris and secrete cytokines and growth factors that may enhance wound healing [26]. To our knowledge, we have proved the presence of adipocytes within human granulation tissue for the first time. Previous studies in murine models have suggested that ASC might differentiate into adipocytes within the injured skin and that a lack of wound-tissue adipocytes results in decreased fibroblast recruitment to the site of injury, with subsequent deficiencies in extracellular matrix remodeling [2]. Of note, the frequency and organization of adipocytes within our granulation tissue specimens were variable depending on patient-specific parameters such as wound type, anatomical origin, and wound age. Our observation requires further validation in a larger patient cohort to gain insight into the abundance of single and clustered adipocytes in wounds of varying aetiologies. However, based on the observed pattern of adipocytes within granulation tissue, and our finding that cells with adipogenic potential reside in granulation tissue, we speculate that adipocyte presence in granulation tissue is a result of migration and differentiation of regenerative progenitor cells.

Given that macrophages, through their transition from a pro- to anti-inflammatory phenotype, are key regulators of the wound healing process [27], we focused on identifying whether or not they play a role in stimulating adipogenesis within granulation tissue. Indeed, analysis of our tissue specimens demonstrated dense clusters of CD68^+^ macrophages immediately adjacent to the adipocytes seen within granulation tissue. By mimicking these conditions with distinct in vitro approaches, we observed that pro-inflammatory M^(IFNG/LPS)^-CM significantly inhibited the ability of ASC to differentiate into mature, lipid-laden adipocytes. This is in agreement with previous reports indicating the attenuating effects on adipocyte differentiation of macrophage-like CM in both murine and human in vitro models [28–30]. However, our observation that this phenotype depends on a pro-and anti-inflammatory stimulation of macrophages is considerably new. A recent study investigating the adipogenic potential of fibro-adipogenic muscle progenitor cells isolated from patients with muscular dystrophy supported our findings by demonstrating that IL1B stimulated macrophages inhibited adipogenesis of these cells [31].

Of note, in our experimental setting, M^(IFNG/LPS)^-CM did not completely attenuate adipocyte differentiation. Rather, M^(IFNG/LPS)^-CM induces a phenotype characterized by numerous small lipid droplets incapable of fusing to larger droplets or budding from endoplasmic reticulum [32]. Whether this phenotype bears some resemblance to the beige or brown adipocytes remains elusive and outside the scope of our current work.

Our analyses on the expression of key regulators of glycolysis and endogenous fatty acid synthesis by M^(IFNG/LPS)-^CM have—to our knowledge—not been addressed by other studies in detail, whereas the effect of single pro-inflammatory cytokines like IL1B or TNFA on glucose uptake, insulin sensitivity, and lipogenesis have been extensively addressed [33]. For example, IL1B inhibits GLUT4 translocation, decreases expression of insulin receptor substrate 1 (IRS1), and induces lipolysis in adipocytes [34], but stimulates triglyceride and cholesterol synthesis in murine hepatic cells [35]. We found that pro-inflammatory cytokines in M^(IFNG/LPS)^-CM induced a shift from endogenous fatty acid synthesis towards glycolysis dominated metabolism, as suggested from protein expression data. Of note, GLUT4 expression and the levels of hexokinases (HK1 and HK2) were reduced by M^(IFNG/LPS)^-CM treatment, whereas all other glycolysis regulating enzymes were induced. M^(IFNG/LPS)^ CM-treated cells might use glutamine as an energy source to bypass limited endogenous glucose levels or favor activation of the pentose phosphate pathway (PPP) [36], as observed in cancer cells, to enable cellular survival under metabolic stress. However, mitochondrial activity and cell proliferation (differentiation preceding mitotic clonal expansion) seemed unaffected by these metabolic changes, but impaired endogenous fatty acid synthesis might be causative for impaired adipocyte differentiation.

Our data further suggested that limited fatty acid synthesis correlated with induction of a possibly non-functional CEBPB isoform, which failed to stimulate downstream PPARG and CEBPA. It is important to note that the anti-adipogenic effect of M^(IFNG/LPS)-^CM occurred in the presence of in vitro adipogenesis inducing agents such as insulin, the CEBPB inducers dexamethasone and IBMX, and the PPARG agonist troglitazone. Somewhat unexpectedly, transgenic PPARG, which was demonstrated to compensate for CEBPA-loss in mouse fibroblasts [37], failed to rescue the anti-adipogenic phenotype. While PPARG overexpression enhanced adipocyte differentiation in control cells, it was almost completely degraded in M^(IFNG/LPS)-^CM and also failed to induce CEBPA, a mechanism essential for adipocyte differentiation [7]. Our observations suggested that pro-inflammatory macrophages not only dampen CEBPB activity, but also compromised PPARG activity by inciting its active degradation. This is complementary to previous research demonstrating that IFNG-treatment leads to increased targeting of PPARG to the ubiquitin proteasome pathway for degradation in adipocytes [38].

A useful method to dampen the anti-adipogenic effect of M^(IFNG/LPS)-^CM was simultaneous treatment of macrophages during their activation phase with NCT. NCT is known to antagonize the inflammatory response of neutrophils and macrophages [39, 40] and has been therefore tested in clinical trials for the treatment of inflammatory diseases and wound healing deficiencies [41]. Interestingly, IL1B was one of the top candidate cytokines downregulated by NCT in IFNG/LPS-treated macrophages. However, blocking of IL1-signaling by addition of IL1RA in differentiating cells was insufficient to antagonize the anti-adipogenic effect of M^(IFNG/LPS)^-CM.

## Conclusions

In conclusion, we demonstrated that inflammatory, but not anti-inflammatory macrophages, dampen adipocyte differentiation by impeding endogenous fatty acid synthesis. Clinically, we propose that the appearance of adipocytes in wound tissue is suggestive of a prevailing anti-inflammatory environment and may be associated with improved healing outcomes.

## Supplementary Information

**Additional file 1: Supplement Figure 1.** Detection of adipocytes in unfixed granulation tissue. Intra-vital confocal microscopy single channel images of unfixed granulation tissue compared with subcutaneous fat tissue (merge displayed in Fig. 1b) stained with BODIPY-493/503 to highlight lipid droplets, wheat germ agglutinin (CF®555 WGA) to highlight ECM substrates and cell morphology (N-acetyl-D-glucosamine and sialic acid present in hyaluronan and other heterogeneous polysaccharides), and Hoechst 33342 staining for nuclei. **Supplement Figure 2.** PPARG overexpression in day 3 differentiated cells. Representative immunoblotting of PPARG- or puromycin overexpressing human ASC subjected to in vitro differentiation for 3 days in the presence of macrophage-CM. **Supplement Figure 3.** Numbers of NCT-regulated cytokines. Comparison of cytokines with greater than two-fold change in response to NCT-treatment in various CM. Shown are the total number of regulated cytokines per CM (Set Size), and the number of regulated cytokines found exclusively in one CM or shared between different CM types (Intersection Size). Intersections between different CM types are indicated with connected black dots, single dots indicate a single CM. As shown, 22, 7, 6 and 2 cytokines were found exclusively regulated by M(IFNG/LPS), Mo, M0 and M(IL4/IL13) respectively. Four cytokines were similarly regulated in M(IFNG/LPS) and Mo, 3 in M(IFNG/LPS) and M0, 2 in M0 and M(IL4/IL13), 1 in M(IFNG/LPS) and M(IL4/IL13), and 2 in M(IFNG/LPS), M0 and M(IL4/IL13). **Supplement Figure 4.** Efficacy of IL1RA to inhibit anti-adipogenic effect of IL1B. IL1B receptor mediated signaling was inhibited by the addition of 100ng/ml recombinant IL1RA. Effect on adipocyte differentiation was evaluated by flow cytometry assessing numbers of lipid-laden cells. Values depict the percentage of lipid-laden adipocytes as a percentage of untreated control cells (0ng IL1B) that emit green fluorescence after staining with BODIPY-493/503, n=3. Statistical significance was determined by using non parametric unpaired Student’s *t*-test. Data are shown as mean ± SEM. Asterisks indicate p-values < 0.05 (*). **Supplement Table 1.** Table with log2 cytokine protein expression levels used to generate the heatmap in Fig. 2c. (1) and (2) depict values obtained from two independent measurements. **Supplement Table 2.** Regulation of cytokines by NCT co-treatment in various CM. Contains log2 fold change (M) and log2 average (A) values representing the extent of regulation, and average expression, of cytokines following NCT co-treatment of CM (e.g. a comparison of NCT co-treated Mo cells against untreated Mo cells). **Supplement Table 3.** List of Primers used for quantitative RT-PCR.

## Data Availability

The datasets supporting the conclusions of this article are included within the article and its additional files.

## Abbreviations

ASC: Adipose-derived stem cells
CM: Conditioned medium
IBMX: 3-Isobutyl-1-methyl-xanthine
LPS: Lipopolysaccharide
M^(IFNG/LPS)^: IFNG/LPS-stimulated macrophages
M^(IL4/IL13)^: IFNG/LPS-stimulated macrophages
NCT: N-chlorotaurine
SVF: Stromal vascular fraction
