# Supplementary material for: Oxidant therapy improves adipogenic differentiation of adipose-derived stem cells in human wound healing
Source: Stem Cell Res Ther. 2021 May 10;12:280. doi: 10.1186/s13287-021-02336-3 (PMC8111898; doi:10.1186/s13287-021-02336-3)
Supplement: Supplementary file 1 — Additional file 1: Supplement Figure 1. Detection of adipocytes in unfixed granulation tissue. Intra-vital confocal microscopy single channel images of unfixed granulation tissue compared with subcutaneous fat tissue (merge displayed in Fig. 1b) stained with BODIPY-493/503 to highlight lipid droplets, wheat germ agglutinin (CF®555 WGA) to highlight ECM substrates and cell morphology (N-acetyl-D-glucosamine and sialic acid present in hyaluronan and other heterogeneous polysaccharides), and Hoechst 33342 staining for nuclei. Supplement Figure 2. PPARG overexpression in day 3 differentiated cells. Representative immunoblotting of PPARG- or puromycin overexpressing human ASC subjected to in vitro differentiation for 3 days in the presence of macrophage-CM. Supplement Figure 3. Numbers of NCT-regulated cytokines. Comparison of cytokines with greater than two-fold change in response to NCT-treatment in various CM. Shown are the total number of regulated cytokines per CM (Set Size), and the number of regulated cytokines found exclusively in one CM or shared between different CM types (Intersection Size). Intersections between different CM types are indicated with connected black dots, single dots indicate a single CM. As shown, 22, 7, 6 and 2 cytokines were found exclusively regulated by M(IFNG/LPS), Mo, M0 and M(IL4/IL13) respectively. Four cytokines were similarly regulated in M(IFNG/LPS) and Mo, 3 in M(IFNG/LPS) and M0, 2 in M0 and M(IL4/IL13), 1 in M(IFNG/LPS) and M(IL4/IL13), and 2 in M(IFNG/LPS), M0 and M(IL4/IL13). Supplement Figure 4. Efficacy of IL1RA to inhibit anti-adipogenic effect of IL1B. IL1B receptor mediated signaling was inhibited by the addition of 100ng/ml recombinant IL1RA. Effect on adipocyte differentiation was evaluated by flow cytometry assessing numbers of lipid-laden cells. Values depict the percentage of lipid-laden adipocytes as a percentage of untreated control cells (0ng IL1B) that emit green fluorescence after staining with BODIPY-493/503, n [file 13287_2021_2336_MOESM1_ESM.docx]

**Supporting Information for:**

**Oxidant therapy improves adipogenic differentiation of adipose derived stem cells in human wound healing**

C. Ploner^1*^, T. Rauchenwald ^1^, C.E. Connolly ^1^, K. Joehrer^2^ , J. Rainer^3^, C. Seifarth^4^, M. Hermann^5^, M. Nagl^6^, S. Lobenwein^1^, D. Wilflingseder^6^, G. Cappellano^1**^, E.M. Morandi^1^ and G. Pierer^1^

^1^ Department of Plastic, Reconstructive and Aesthetic Surgery, Medical University of Innsbruck, Austria

^2^ Tyrolean Cancer Research Institute, Innsbruck, Austria

^3^ Institute for Biomedicine, Eurac Research, Affiliated Institute of the University of Lübeck, Bolzano, Italy

^4^ Department of Ophthalmology, Medical University of Innsbruck, Innsbruck, Austria

^5^ Department of Anesthesiology and Critical Care Medicine, Medical University of Innsbruck, Austria

^6^ Institute of Hygiene and Medical Microbiology, Medical University of Innsbruck, Innsbruck, Austria

* corresponding author

Table of contents:

1. Supplement Figure 1
2. Supplement Figure 2
3. Supplement Figure 3
4. Supplement Figure 4
5. Supplement Table 1
6. Supplement Table 2
7. Supplement Table 3
8. Supplemental Information: Full Blot Images of WB in the manuscript

***Supplement Figure 1:* Detection of adipocytes in unfixed granulation tissue.** Intra-vital confocal microscopy single channel images of unfixed granulation tissue compared with subcutaneous fat tissue (merge displayed in Fig. 1B) stained with BODIPY-493/503 to highlight lipid droplets, wheat germ agglutinin (CF®555 WGA) to highlight ECM substrates and cell morphology (N-acetyl-D-glucosamine and sialic acid present in hyaluronan and other heterogeneous polysaccharides), and Hoechst 33342 staining for nuclei.


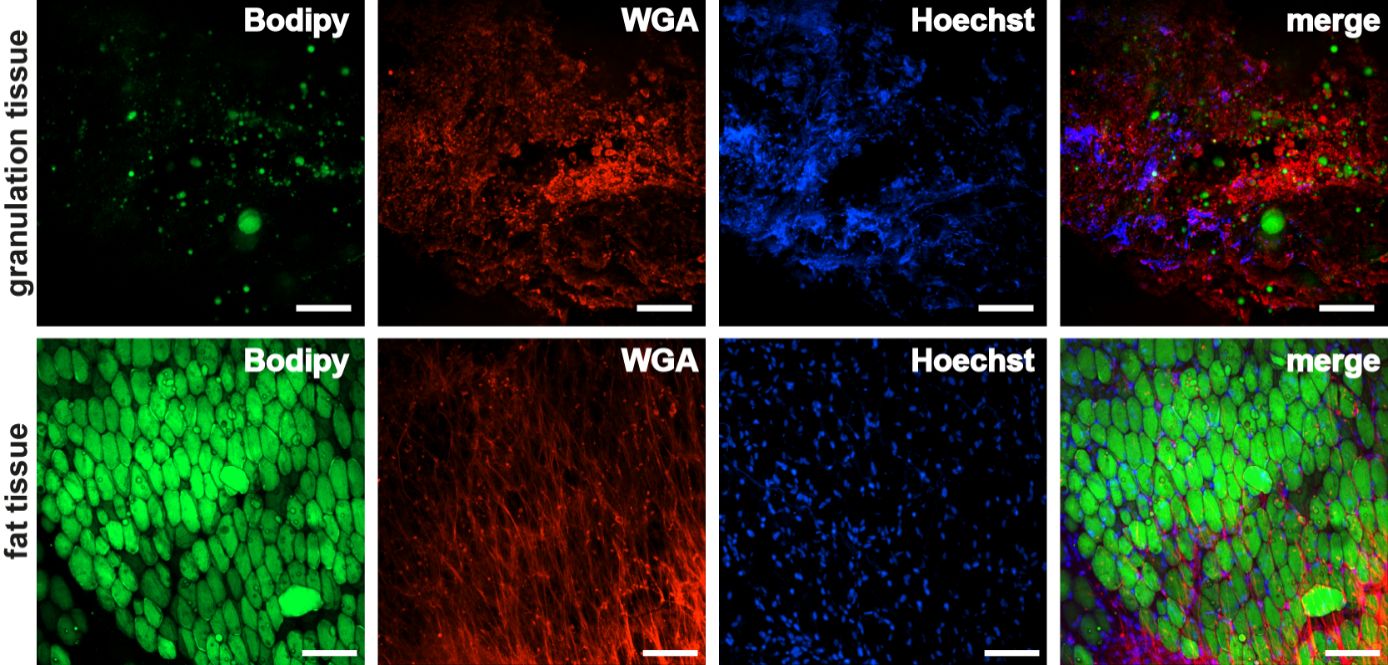


***Supplement Figure 2:* PPARG overexpression in day 3 differentiated cells.** Representative immunoblotting of PPARG- or puromycin overexpressing human ASC subjected to in-vitro differentiation for 3 days in the presence of macrophage-CM.


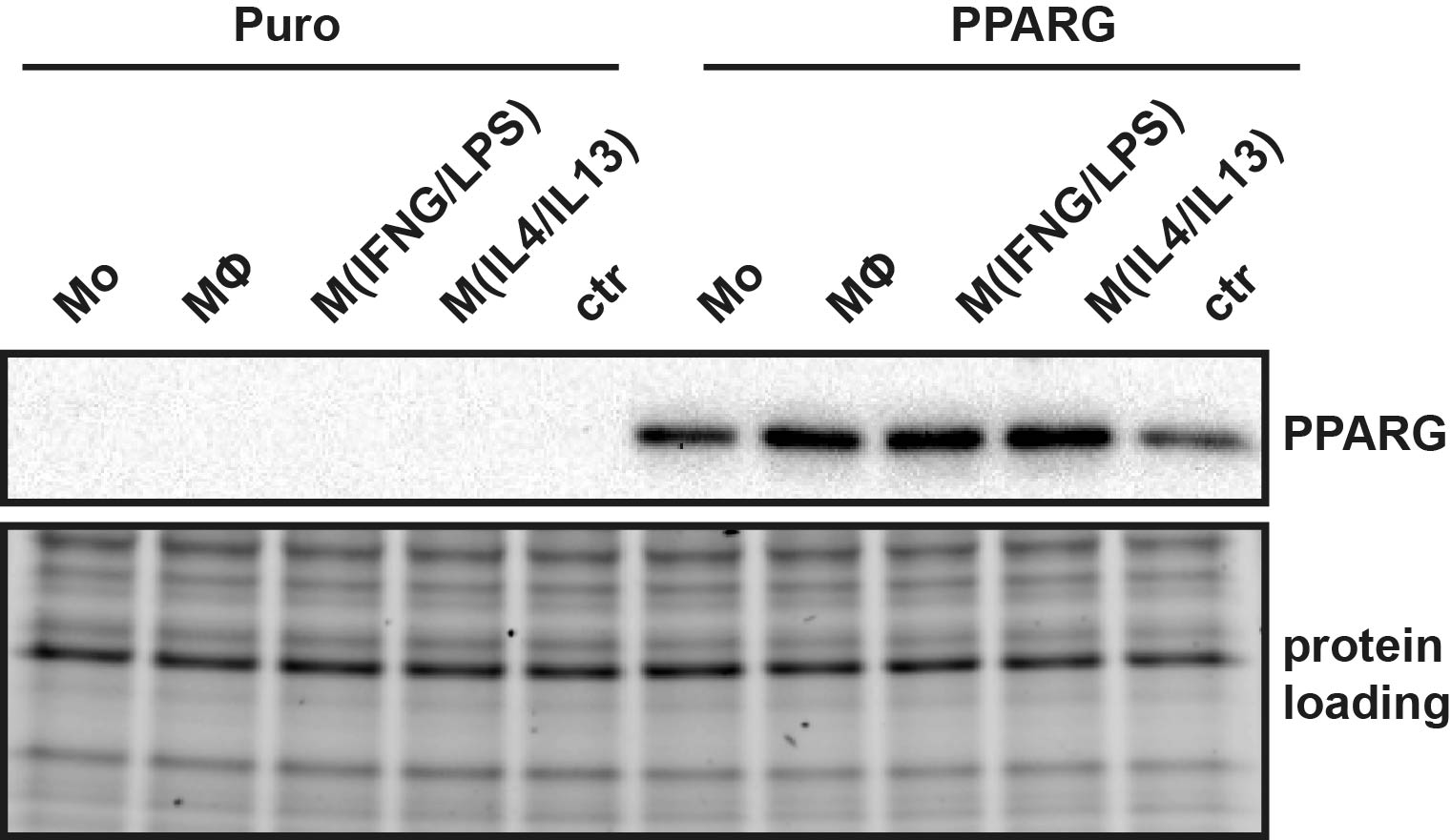


***Supplement Figure 3:* Numbers of NCT-regulated cytokines.** Comparison of cytokines with greater than two-fold change in response to NCT-treatment in various CM. Shown are the total number of regulated cytokines per CM (Set Size), and the number of regulated cytokines found exclusively in one CM or shared between different CM types (Intersection Size). Intersections between different CM types are indicated with connected black dots, single dots indicate a single CM. As shown, 22, 7, 6 and 2 cytokines were found exclusively regulated by M(IFNG/LPS), Mo, M0 and M(IL4/IL13) respectively. Four cytokines were similarly regulated in M(IFNG/LPS) and Mo, 3 in M(IFNG/LPS) and M0, 2 in M0 and M(IL4/IL13), 1 in M(IFNG/LPS) and M(IL4/IL13), and 2 in M(IFNG/LPS), M0 and M(IL4/IL13).

***
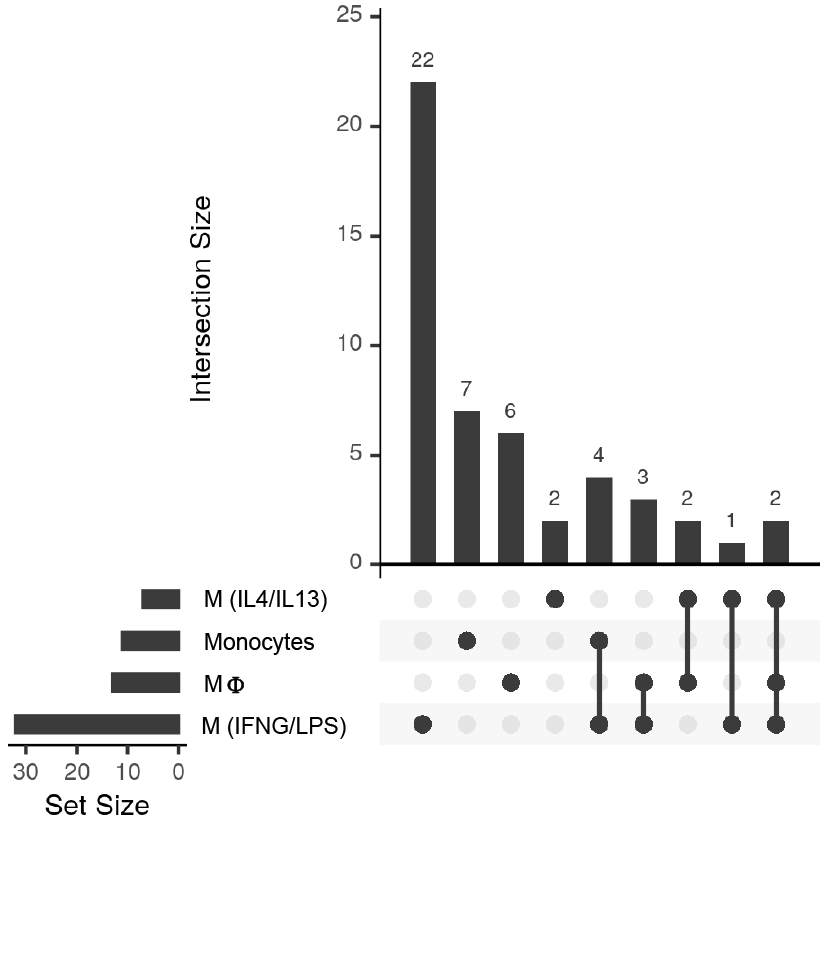
***

***Supplement Figure 4:* Efficacy of IL1RA to inhibit anti-adipogenic effect of IL1B.** IL1B receptor mediated signaling was inhibited by the addition of 100ng/ml recombinant IL1RA. Effect on adipocyte differentiation was evaluated by flow cytometry assessing numbers of lipid-laden cells. Values depict the percentage of lipid-laden adipocytes as a percentage of untreated control cells (0ng IL1B) that emit green fluorescence after staining with BODIPY-493/503, n=3. Statistical significance was determined by using non parametric unpaired Student’s t-test. Data are shown as mean ± SEM. Asterisks indicate p-values < 0.05 (*).

***
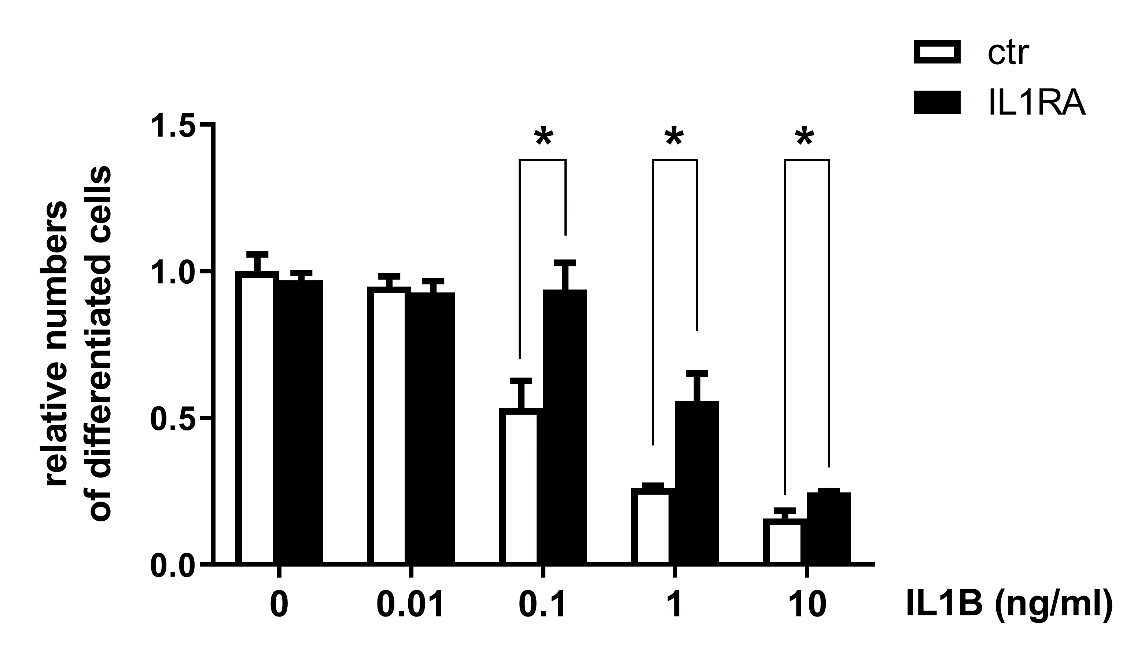
***

***Supplement Table 1.***

*Table with log2 cytokine protein expression levels used to generate the heatmap in Figure 2C.*

*(1) and (2) depict values obtained from two independent measurements.*

| **cytokine** | **Mo (1)** | **Mo (2)** | **MΦ (1)** | **MΦ (2)** | **M(IFNG/LPS) (1)** | **M(IFNG/LPS) (2)** | **M(IL4/IL13) (1)** | **M(IL4/IL13) (2)** |
| --- | --- | --- | --- | --- | --- | --- | --- | --- |
| CXCL5 | 7.2 | 7.5 | 7.7 | 6.8 | 10.8 | 9.5 | 7.5 | 7.4 |
| CSF3 | 8.0 | 8.1 | 9.1 | 8.2 | 10.9 | 10.2 | 8.7 | 8.5 |
| CSF2 | 7.5 | 8.1 | 8.9 | 8.1 | 11.1 | 10.2 | 8.5 | 8.5 |
| CXCL1, CXCL2, CXCL3 | 8.2 | 8.5 | 10.1 | 11.0 | 11.9 | 11.3 | 9.3 | 9.4 |
| CXCL1 | 7.5 | 7.3 | 7.9 | 6.7 | 8.6 | 7.8 | 7.5 | 7.2 |
| CCL1 | 7.4 | 6.5 | 11.0 | 11.5 | 11.7 | 11.9 | 11.0 | 11.4 |
| IL1A | 8.0 | 8.3 | 9.1 | 8.1 | 10.9 | 10.1 | 8.5 | 8.7 |
| IL1B | 6.9 | 6.7 | 8.1 | 5.8 | 13.6 | 13.8 | 8.4 | 7.0 |
| IL2 | 8.0 | 8.5 | 9.5 | 8.3 | 11.3 | 10.6 | 9.2 | 8.7 |
| IL3 | 7.8 | 8.1 | 8.4 | 7.3 | 8.5 | 8.5 | 8.2 | 7.9 |
| IL4 | 6.9 | 6.7 | 10.8 | 9.1 | 9.6 | 10.4 | 10.7 | 10.3 |
| IL5 | 8.8 | 8.6 | 9.9 | 8.1 | 11.1 | 10.6 | 9.0 | 7.3 |
| IL6 | 8.1 | 8.3 | 9.3 | 8.2 | 11.9 | 10.4 | 8.8 | 8.4 |
| IL7 | 8.4 | 8.6 | 9.5 | 8.7 | 11.2 | 10.5 | 8.9 | 8.9 |
| IL8 | 8.9 | 8.9 | 10.0 | 9.1 | 10.5 | 10.2 | 9.5 | 9.3 |
| IL10 | 7.9 | 8.3 | 9.0 | 8.4 | 10.8 | 9.9 | 8.5 | 8.4 |
| IL12B | 8.0 | 8.1 | 8.7 | 7.9 | 8.3 | 8.4 | 8.4 | 8.1 |
| IL13 | 8.3 | 8.4 | 9.1 | 8.2 | 10.7 | 10.0 | 12.4 | 13.4 |
| IL15 | 8.7 | 8.6 | 9.2 | 8.4 | 11.1 | 10.5 | 8.8 | 8.7 |
| IFNG | 8.2 | 8.0 | 8.6 | 6.8 | 10.9 | 10.2 | 8.4 | 7.3 |
| CCL2 | 8.4 | 8.8 | 12.3 | 12.4 | 13.7 | 12.1 | 11.3 | 12.9 |
| CCL8 | 7.1 | 7.4 | 9.0 | 9.7 | 14.3 | 14.7 | 12.0 | 10.4 |
| CCL7 | 6.7 | 6.7 | 10.9 | 11.9 | 14.3 | 15.1 | 13.0 | 12.0 |
| CSF1 | 7.1 | 7.2 | 7.7 | 5.7 | 7.6 | 8.2 | 8.0 | 7.9 |
| CCL22 | 8.8 | 9.0 | 10.0 | 9.9 | 9.9 | 8.8 | 11.5 | 11.5 |
| CXCL9 | 8.3 | 8.3 | 9.2 | 8.1 | 11.3 | 10.6 | 8.8 | 8.5 |
| CCL4 | 7.6 | 8.0 | 13.4 | 13.0 | 13.2 | 13.4 | 13.2 | 13.1 |
| CCL15 | 7.0 | 6.7 | 7.2 | 5.2 | 6.9 | 6.9 | 6.9 | 6.8 |
| CCL5 | 15.1 | 14.8 | 15.2 | 14.8 | 15.0 | 15.2 | 15.1 | 14.9 |
| KITLG | 8.5 | 8.6 | 8.8 | 7.8 | 8.5 | 8.9 | 8.5 | 8.3 |
| CXCL12 | 7.2 | 7.0 | 7.4 | 6.4 | 7.3 | 7.6 | 7.4 | 7.2 |
| CCL17 | 7.2 | 7.1 | 8.0 | 6.3 | 7.6 | 7.5 | 8.8 | 8.2 |
| TGFB1 | 8.5 | 8.6 | 9.4 | 8.7 | 11.1 | 10.6 | 8.9 | 9.0 |
| TNFA | 8.8 | 8.8 | 10.1 | 8.7 | 10.8 | 11.1 | 9.6 | 8.9 |
| TNFB | 8.8 | 9.2 | 9.8 | 8.9 | 11.6 | 11.0 | 9.4 | 9.3 |
| EGF | 6.9 | 7.0 | 7.3 | 4.8 | 6.8 | 7.3 | 7.2 | 6.7 |
| IGF1 | 7.0 | 6.9 | 7.5 | 5.3 | 7.1 | 7.3 | 7.3 | 6.9 |
| ANG | 10.7 | 10.0 | 10.7 | 10.0 | 10.3 | 9.6 | 10.3 | 9.4 |
| OSM | 7.7 | 7.5 | 8.0 | 6.6 | 7.8 | 8.1 | 7.8 | 7.3 |
| THPO | 7.4 | 7.2 | 7.7 | 5.5 | 7.9 | 8.0 | 7.4 | 7.0 |
| VEGFA | 8.3 | 8.2 | 9.9 | 8.0 | 9.1 | 8.7 | 10.0 | 9.3 |
| TGFB1 | 7.0 | 6.8 | 7.4 | 5.5 | 7.7 | 7.3 | 7.3 | 7.2 |
| LEP | 8.0 | 8.3 | 8.5 | 8.2 | 8.4 | 8.6 | 8.2 | 8.1 |
| BDNF | 7.1 | 7.0 | 7.5 | 6.1 | 7.4 | 7.3 | 7.1 | 7.0 |
| CXCL13 | 6.9 | 6.7 | 7.2 | 6.0 | 7.9 | 7.1 | 6.9 | 6.8 |
| CCL23 | 8.1 | 7.7 | 7.7 | 6.6 | 7.9 | 7.9 | 11.3 | 12.2 |
| CCL11 | 8.4 | 8.2 | 8.3 | 6.3 | 7.4 | 8.4 | 8.1 | 7.3 |
| CCL24 | 10.0 | 9.5 | 10.1 | 7.1 | 7.8 | 7.7 | 10.0 | 8.2 |
| CCL26 | 7.2 | 7.4 | 8.2 | 6.7 | 7.6 | 8.5 | 8.1 | 7.5 |
| FGF4 | 7.0 | 7.1 | 7.4 | 5.2 | 7.3 | 7.2 | 7.2 | 6.9 |
| FGF6 | 6.9 | 7.0 | 7.2 | 5.5 | 7.0 | 7.2 | 7.0 | 6.7 |
| FGF7 | 7.0 | 6.8 | 7.3 | 5.1 | 7.1 | 7.3 | 7.1 | 6.9 |
| FGF9 | 7.1 | 6.9 | 7.5 | 5.0 | 7.3 | 7.3 | 7.1 | 7.0 |
| FLT3LG | 8.0 | 7.4 | 9.3 | 8.3 | 9.9 | 9.2 | 9.4 | 7.1 |
| CX3CL1 | 7.0 | 7.3 | 11.9 | 11.6 | 11.7 | 11.9 | 11.6 | 11.9 |
| CXCL6 | 7.6 | 6.9 | 7.4 | 6.1 | 9.0 | 9.1 | 7.3 | 7.2 |
| GDNF | 7.2 | 7.4 | 7.7 | 6.4 | 7.3 | 7.6 | 7.3 | 7.2 |
| HGF | 10.2 | 9.3 | 7.3 | 5.3 | 7.1 | 7.1 | 7.0 | 6.8 |
| IGFBP1 | 7.1 | 6.6 | 7.1 | 4.2 | 6.9 | 7.2 | 6.7 | 6.8 |
| IGFBP2 | 11.7 | 10.9 | 8.6 | 6.2 | 7.2 | 7.8 | 8.2 | 7.4 |
| IGFBP3 | 7.4 | 7.6 | 7.8 | 6.3 | 7.9 | 7.8 | 7.7 | 7.6 |
| IGFBP4 | 7.0 | 6.9 | 7.3 | 4.0 | 6.9 | 7.1 | 6.9 | 6.8 |
| IL16 | 6.9 | 6.7 | 7.1 | 4.4 | 7.0 | 7.1 | 7.0 | 6.7 |
| CXCL10 | 8.1 | 8.4 | 8.8 | 8.0 | 12.0 | 12.1 | 8.2 | 7.6 |
| LIF | 8.7 | 9.5 | 9.8 | 7.9 | 9.4 | 9.6 | 9.6 | 9.5 |
| TNFSF14 | 7.2 | 7.2 | 8.0 | 8.4 | 9.2 | 9.1 | 7.4 | 7.7 |
| CCL13 | 7.5 | 7.2 | 7.6 | 6.0 | 8.7 | 8.4 | 10.9 | 8.7 |
| MIF | 12.1 | 12.0 | 11.2 | 10.7 | 11.8 | 11.5 | 11.5 | 11.5 |
| CCL20 | 7.5 | 7.1 | 14.2 | 13.8 | 14.0 | 14.3 | 13.9 | 14.0 |
| PPBP | 7.0 | 6.6 | 7.5 | 7.3 | 7.8 | 7.9 | 7.0 | 7.1 |
| NTF3 | 7.5 | 7.5 | 8.0 | 6.4 | 7.5 | 7.6 | 7.5 | 7.4 |
| NTF4 | 7.8 | 7.7 | 8.0 | 7.0 | 7.9 | 8.2 | 7.8 | 7.6 |
| SPP1 | 7.6 | 7.2 | 13.0 | 13.3 | 12.4 | 12.7 | 12.9 | 12.7 |
| TNFRSF11B | 7.5 | 6.9 | 7.8 | 7.4 | 7.2 | 7.5 | 8.0 | 7.2 |
| CCL18 | 7.6 | 7.4 | 8.2 | 6.4 | 7.8 | 8.5 | 7.9 | 7.4 |
| PIGF | 7.5 | 7.7 | 7.8 | 6.9 | 8.1 | 8.0 | 7.7 | 7.6 |
| TGFB2 | 10.7 | 10.5 | 10.8 | 9.9 | 10.9 | 10.9 | 10.5 | 10.2 |
| TGFB3 | 7.0 | 6.8 | 7.3 | 4.6 | 7.1 | 7.2 | 6.9 | 7.0 |
| TIMP1 | 12.4 | 12.4 | 12.7 | 11.9 | 12.4 | 12.6 | 12.5 | 12.5 |
| TIMP2 | 13.9 | 13.8 | 13.9 | 13.3 | 13.3 | 13.1 | 13.7 | 13.8 |

***Supplement Table 2.***

*Regulation of cytokines by NCT co-treatment in various CM. Contains log2 fold change (M) and log2 average (A) values representing the extent of regulation, and average expression, of cytokines following NCT co-treatment of CM (e.g. a comparison of NCT co-treated Mo cells against untreated Mo cells).*

| **cytokine** | **M.**  **Mo** | **M.**  **MΦ** | **M.**  **M(IFNG/LPS)** | **M.**  **M (IL4/IL13)** | **A.**  **Mo** | **A.**  **MΦ** | **A.**  **M(IFNG/LPS)** | **A.**  **M(IL4/IL13)** |
| --- | --- | --- | --- | --- | --- | --- | --- | --- |
| CXCL5 | 0.9 | 0.9 | 0.2 | 0.4 | 6.4 | 6.3 | 10.8 | 6.2 |
| CSF3 | -0.2 | 0.2 | 1.8 | 0.5 | 7.6 | 8.0 | 9.7 | 8.5 |
| CSF2 | -0.2 | 0.0 | 2.1 | 0.8 | 7.8 | 8.1 | 9.6 | 8.4 |
| CXCL1, CXCL2, CXCL3 | -0.2 | 0.7 | 0.7 | 0.3 | 8.3 | 10.6 | 11.9 | 10.5 |
| CXCL1 | -0.4 | 0.5 | 2.1 | 0.6 | 6.2 | 6.5 | 7.7 | 6.8 |
| CCL1 | 0.3 | 4.0 | 0.5 | 2.8 | 2.8 | 9.5 | 11.7 | 10.3 |
| IL1A | -0.1 | 0.0 | 1.9 | 0.6 | 7.8 | 8.1 | 9.6 | 8.5 |
| IL1B | -0.8 | 2.1 | 2.5 | 0.6 | 4.2 | 4.8 | 11.0 | 6.1 |
| IL2 | -0.3 | 0.2 | 2.3 | 1.0 | 7.9 | 8.2 | 9.9 | 8.6 |
| IL3 | -0.4 | 0.3 | 0.9 | 1.0 | 6.9 | 7.1 | 8.4 | 7.5 |
| IL4 | -1.3 | -0.2 | -1.0 | -0.7 | 5.0 | 9.2 | 9.4 | 10.0 |
| IL5 | -0.5 | 0.8 | 1.8 | 0.7 | 7.8 | 7.7 | 10.1 | 9.0 |
| IL6 | -0.3 | 0.8 | 2.3 | 0.4 | 7.6 | 7.8 | 10.0 | 8.5 |
| IL7 | -0.4 | 0.4 | 1.9 | 0.5 | 8.2 | 8.5 | 10.1 | 8.9 |
| IL8 | -0.6 | -0.1 | 0.7 | 0.2 | 8.6 | 9.2 | 10.1 | 9.4 |
| IL10 | -0.5 | 0.1 | 2.0 | 0.5 | 7.8 | 8.4 | 9.7 | 8.6 |
| IL12B | 0.1 | 0.1 | 0.2 | -0.1 | 7.5 | 7.8 | 8.1 | 8.0 |
| IL13 | -0.3 | 0.1 | 1.8 | -1.3 | 7.8 | 8.1 | 9.6 | 12.1 |
| IL15 | -0.5 | 0.1 | 1.9 | 0.5 | 8.1 | 8.3 | 9.8 | 8.7 |
| IFNG | -0.3 | 1.0 | 2.0 | 0.8 | 6.4 | 6.3 | 9.6 | 7.6 |
| CCL2 | -0.7 | 1.9 | 1.9 | 1.7 | 8.7 | 11.5 | 12.3 | 11.8 |
| CCL8 | 0.2 | 3.6 | -0.1 | 1.3 | 5.7 | 7.9 | 14.4 | 9.3 |
| CCL7 | 0.1 | 1.3 | -0.2 | -0.6 | 5.1 | 11.2 | 14.5 | 13.2 |
| CSF1 | -1.5 | -0.6 | 1.2 | -0.3 | 6.2 | 6.0 | 7.8 | 6.8 |
| CCL22 | 1.8 | 0.9 | 1.1 | 0.4 | 8.6 | 9.4 | 9.7 | 11.9 |
| CXCL9 | -0.1 | 0.8 | 2.1 | 0.4 | 7.5 | 7.7 | 9.8 | 8.5 |
| CCL4 | -1.2 | 0.2 | -0.2 | -0.1 | 7.5 | 12.9 | 13.5 | 13.3 |
| CCL15 | -0.3 | 1.2 | 1.0 | -0.1 | 4.7 | 4.6 | 4.8 | 4.8 |
| CCL5 | 0.8 | 0.2 | -0.2 | -0.2 | 14.5 | 14.6 | 14.9 | 14.8 |
| KITLG | -0.1 | 0.1 | 0.1 | 0.2 | 7.6 | 7.7 | 8.1 | 7.9 |
| CXCL12 | 0.4 | 0.5 | 0.7 | 0.8 | 6.3 | 6.1 | 6.9 | 7.0 |
| CCL17 | 0.1 | 0.3 | 0.8 | 2.5 | 6.5 | 6.2 | 6.9 | 7.4 |
| TGFB1 | 0.0 | 0.8 | 1.9 | 0.6 | 8.3 | 8.3 | 10.0 | 8.8 |
| TNFA | -0.6 | -0.3 | 1.2 | 0.1 | 8.7 | 8.8 | 10.2 | 9.3 |
| TNFB | -0.3 | 0.3 | 2.1 | 0.5 | 8.6 | 8.7 | 10.3 | 9.2 |
| EGF | -0.7 | -0.3 | -0.4 | -0.3 | 5.6 | 5.0 | 6.2 | 5.7 |
| IGF1 | -1.4 | -0.8 | 0.3 | 0.7 | 5.8 | 5.7 | 6.6 | 6.3 |
| ANG | 0.8 | 0.1 | -0.1 | -0.1 | 10.8 | 9.9 | 10.4 | 10.4 |
| OSM | 0.4 | 0.5 | 0.4 | 0.2 | 6.2 | 6.3 | 7.1 | 7.1 |
| THPO | -0.3 | 0.0 | 1.0 | 0.1 | 5.2 | 5.5 | 6.9 | 6.3 |
| VEGFA | 0.3 | 0.7 | -0.3 | 0.2 | 7.3 | 7.7 | 8.7 | 8.8 |
| TGFB1 | 0.1 | -0.1 | 0.7 | 0.6 | 5.6 | 5.6 | 5.7 | 5.2 |
| LEP | 0.2 | 0.2 | 0.3 | 0.2 | 8.2 | 8.1 | 8.6 | 8.3 |
| BDNF | 0.1 | 0.9 | 0.9 | 0.3 | 5.6 | 5.6 | 6.4 | 5.8 |
| CXCL13 | -0.4 | 0.9 | 2.1 | 0.1 | 5.0 | 5.5 | 5.8 | 5.6 |
| CCL23 | 0.3 | 0.5 | 1.1 | -0.4 | 7.2 | 6.3 | 7.7 | 10.6 |
| CCL11 | 0.0 | -0.5 | -0.8 | -0.9 | 7.7 | 6.6 | 7.1 | 7.0 |
| CCL24 | 0.5 | 1.0 | 0.4 | 0.4 | 7.7 | 6.6 | 6.7 | 6.9 |
| CCL26 | -1.5 | -0.5 | 0.5 | -0.1 | 6.5 | 7.0 | 7.9 | 7.6 |
| FGF4 | 0.3 | 0.5 | 1.1 | 0.9 | 5.3 | 4.9 | 6.0 | 5.7 |
| FGF6 | -0.6 | 0.6 | 1.1 | 0.7 | 5.0 | 5.2 | 5.4 | 5.1 |
| FGF7 | 0.4 | 1.0 | 0.6 | 1.0 | 5.6 | 4.6 | 5.8 | 5.6 |
| FGF9 | -0.2 | 0.0 | 0.5 | 0.6 | 5.1 | 5.0 | 5.7 | 5.5 |
| FLT3LG | 0.1 | 3.7 | 1.1 | 0.4 | 5.3 | 6.5 | 9.6 | 8.0 |
| CX3CL1 | -0.6 | 0.2 | 0.1 | 0.1 | 6.0 | 11.5 | 12.0 | 11.9 |
| CXCL6 | -0.4 | 1.0 | 1.3 | -0.4 | 4.5 | 5.6 | 9.1 | 5.9 |
| GDNF | -0.1 | 0.1 | 0.8 | -0.2 | 6.4 | 6.4 | 6.6 | 6.5 |
| HGF | 2.1 | -0.4 | 0.3 | -0.7 | 8.5 | 5.5 | 5.3 | 4.8 |
| IGFBP1 | 1.9 | -0.2 | 0.3 | 0.4 | 3.3 | 4.3 | 5.0 | 4.3 |
| IGFBP2 | 0.9 | -1.4 | -0.9 | 0.1 | 10.9 | 6.9 | 6.2 | 6.3 |
| IGFBP3 | -1.1 | 1.0 | 1.5 | 0.4 | 5.5 | 5.8 | 6.5 | 5.1 |
| IGFBP4 | -0.2 | -0.7 | 0.8 | -0.5 | 4.9 | 4.4 | 4.7 | 4.8 |
| IL16 | 1.2 | 0.3 | 0.4 | 0.1 | 4.0 | 4.3 | 4.9 | 4.9 |
| CXCL10 | 0.9 | 1.3 | 0.0 | 0.2 | 7.6 | 7.4 | 11.8 | 7.5 |
| LIF | -0.3 | -0.1 | 0.7 | 0.2 | 8.3 | 8.0 | 8.6 | 8.3 |
| TNFSF14 | -0.5 | 1.3 | 0.0 | 0.6 | 5.7 | 7.8 | 9.7 | 8.3 |
| CCL13 | 0.1 | 0.3 | 1.0 | 0.2 | 5.9 | 5.9 | 7.3 | 7.6 |
| MIF | 0.0 | -0.4 | 0.3 | 0.4 | 12.0 | 10.9 | 11.0 | 10.8 |
| CCL20 | 0.0 | 1.2 | -0.1 | 0.9 | 6.8 | 13.2 | 14.1 | 13.6 |
| PPBP |  | -0.3 | -0.4 | -0.4 |  | 7.4 | 9.6 | 7.8 |
| NTF3 | 0.3 | 0.2 | -0.1 | 0.2 | 6.2 | 6.3 | 6.5 | 6.4 |
| NTF4 | 0.3 | 0.3 | 0.5 | 0.5 | 6.6 | 6.8 | 7.3 | 7.2 |
| SPP1 | -2.9 | 0.4 | 0.1 | 0.2 | 7.7 | 13.1 | 13.3 | 13.5 |
| TNFRSF11B | 0.2 | 2.4 | 1.6 | 2.7 | 5.1 | 6.2 | 7.4 | 8.0 |
| CCL18 | -0.4 | 0.3 | 0.3 | -0.5 | 6.9 | 6.3 | 7.7 | 7.0 |
| PIGF | -0.1 | 0.1 | 1.2 | 0.1 | 6.8 | 6.8 | 7.5 | 7.1 |
| TGFB2 | 0.0 | -0.1 | 0.7 | 0.0 | 10.4 | 9.9 | 10.5 | 10.2 |
| TGFB3 | -0.6 | -0.9 | 1.4 | 0.9 | 5.1 | 5.0 | 4.8 | 4.9 |
| TIMP1 | -0.2 | -0.1 | -0.1 | 0.2 | 12.3 | 12.0 | 12.1 | 12.2 |
| TIMP2 | 0.4 | 0.2 | 0.2 | 0.3 | 13.6 | 13.2 | 13.1 | 13.5 |

***Supplement Table 3.***

*List of Primers used for quantitative RT-PCR.*

| Gene | Ref.Seq. | Sense primer (5’-3’) | Antisense primer (5’-3’) |
| --- | --- | --- | --- |
| human ACC | NM_198834 | CATATTGAGGATGACAGGCTGG | CTCATAGTTGACCTGCTTTCTG |
| human ADIPOQ | NM_004797 | gatggcagagatggcaccc | GGAATTTACCAGTGGAGCCA |
| human CCL2 | NM_002982 | GTCTTGAAGATCACAGCTTCTTTG | AGCCAGATGCAATCAATGCC |
| human CCL5 | NM_001278736 | GAGGCTTCCCCTCACTATCC | CTCAAGTGATCCACCCACCT |
| human CD200R1 | NM_138806 | GAGCAATGGCACAGTGACTGTT | GTGGCAGGTCACGGTAGACA |
| human CD80 | NM_005191 | CTGCCTGACCTACTGCTTTG | GGCGTACACTTTCCCTTCTC |
| human CEBPA | NM_004364 | TGGACAAGAACAGCAACGAGTA | ATTGTCACTGGTCAGCTCCAG |
| human CEBPB | NM_005194 | GACAAGCACAGCGACGAGTA | AGCTGCTCCACCTTCTTCTG |
| human CEBPD | NM_005195 | CCATGTACGACGACGAGAG | GTGATTGCTGTTGAAGAGGTCG |
| human CIDEC | NM_001199623 | GGGATGAGAAACATGGAGTCCAAC | CACAGAGGTACGCACTGACAC |
| human CXCL8 | NM_000584 | ATGACTTCCAAGCTGGCCGTGGCT | TCTCAGCCCTCTTCAAAAACTTCTC |
| human DGAT2 | NM_032564 | AGGGGTCTGGGAGATGGG | GCCAGGTGACAGAGAAGAGG |
| human ELOVL3 | NM_152310 | GCTACTTACCGGGGGCCTAA | TGAAGGCTGTGTCTCCGAGTT |
| human ELOVL6 | NM_24090 | CTGGTCTCTGACCCTTGCAG | TGCTTCAGGCCTTTGGTCAT |
| human FABP4 | NM_001442 | TCAGTGTGAATGGGGATGTGATC | TCAACGTCCCTTGGCTTATGC |
| human FASN | NM_004104 | CATCCAGATAGGCCTCATAGA | CTCCATGAAGTAGGAGTGGAA |
| human FGF2 | NM_002006 | GGCTTCTTCCTGCGCATCCAC | GGTAACGGTTAGCACACACTCC |
| human GAPDH | NM_002046 | CAACGAATTTACAGCA | TGTGAGGAGGATTCAG |
| human CXCL8 | NM_000584 | ATGACTTCCAAGCTGGCCGTGGCT | TCTCAGCCCTCTTCAAAAACTTCTC |
| human DGAT2 | NM_032564 | AGGGGTCTGGGAGATGGG | GCCAGGTGACAGAGAAGAGG |
| human ELOVL3 | NM_152310 | GCTACTTACCGGGGGCCTAA | TGAAGGCTGTGTCTCCGAGTT |
| human ELOVL6 | NM_24090 | CTGGTCTCTGACCCTTGCAG | TGCTTCAGGCCTTTGGTCAT |
| human FABP4 | NM_001442 | TCAGTGTGAATGGGGATGTGATC | TCAACGTCCCTTGGCTTATGC |
| human FASN | NM_004104 | CATCCAGATAGGCCTCATAGA | CTCCATGAAGTAGGAGTGGAA |
| human FGF2 | NM_002006 | GGCTTCTTCCTGCGCATCCAC | GGTAACGGTTAGCACACACTCC |
| human GAPDH | NM_002046 | CAACGAATTTACAGCA | TGTGAGGAGGATTCAG |
| human IL1B | NM_000576 | ACAGATGAAGTGCTCCTTCCA | GTCGGAGATTCGTAGCTGGAT |
| human IL6 | NM_000600 | AATTCGGTACATCCTCGACGG | GGTTGTTTTCTGCCAGTGCC |
| human LEPTIN | NM_000230 | CACACGCAGTCAGTCTCCTC | AGGTTCTCCAGGTCGTTGG |
| human NAMPT | NM_005476 | GCAGAAGCCGAGTTCAACAT | TCTGTCTTCTTTTCACGGCA |
| human PDGFA | NM_002607 | CCTGCCCATTCGGAGGAAGAG | TTGGCCACCTTGACGCTGCG |
| human PLIN1 | NM_002666 | GGGAAGAAGTTGAAGCTTGAGGAG | AGGTCTTCTGGAAGCATTCGC |
| human PPARG | NM_005037 | GAGCCCAAGTTTGAGTTTGC | CTGTGAGGACTCAGGGTGGT |
| human RARRES2 | NM_002889 | tggaagaaacccgagtgcaaa | AGAACTTGGGTCTCTATGGGG |
| human RBP4 | NM_006744 | TTCGACAAGGCTCGCTTCTC | CGATGTTGTCCTGCAGAAAGAG |
| human SCD1 | NM_005063 | CACTTGGGAGCCCTGTATGG | TGAGCTCCTGCTGTTATGCC |
| human TGM2 | NM_004613 | GGCCTGTGCCATCAGTATCT | GGAGATCCATCATCTCTCC |
| human VEGFA | NM_003376 | CTACCTCCACCATGCCAAGT | GCAGTAGCTGCGCTGATAGA |
| human 18S rRNA | NR_046261 | GCAATTATTCCCCATGAACG | GGCCTCACTAAACCATCCAA |

**Supplemental Information: Full Blot Images of WB in the manuscript**

**Title**

Adipocyte repopulation of human granulation tissue depends on macrophage polarization

**Authors**

Christian Ploner, Tina Rauchenwald, Catherine E. Connolly, Karin Joehrer , Johannes Rainer, Christoph Seifarth, Martin Hermann, Markus Nagl, Susanne Lobenwein, Doris Wilflingseder, Giuseppe Cappellano, Evi Morandi and Gerhard Pierer

**Description**

In the supplemental information the original uncropped merged images are shown as full length blots. Signals were acquired using a ChemidocMP gel analyzer. Shown are uncropped merged images of the chemiluminescence signal (antibody signal) and white light acquisition (membrane image, sizemarker). Unprocessed images were merged using Image Lab software (Version 5.2.1).

**Full-length unedited images for Fig. 4C**

*
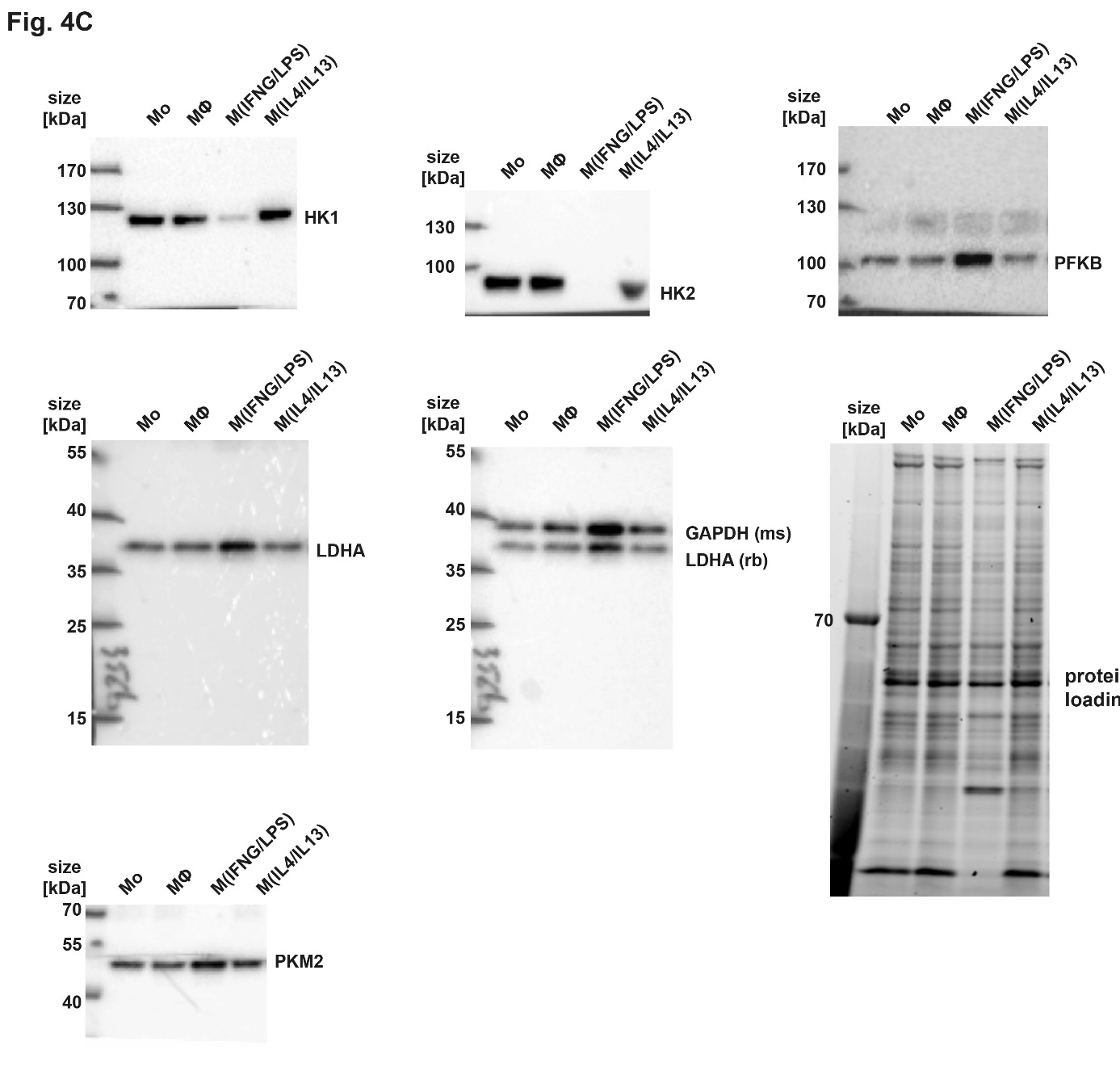
*

**Full-length unedited images for Fig. 4E**

**
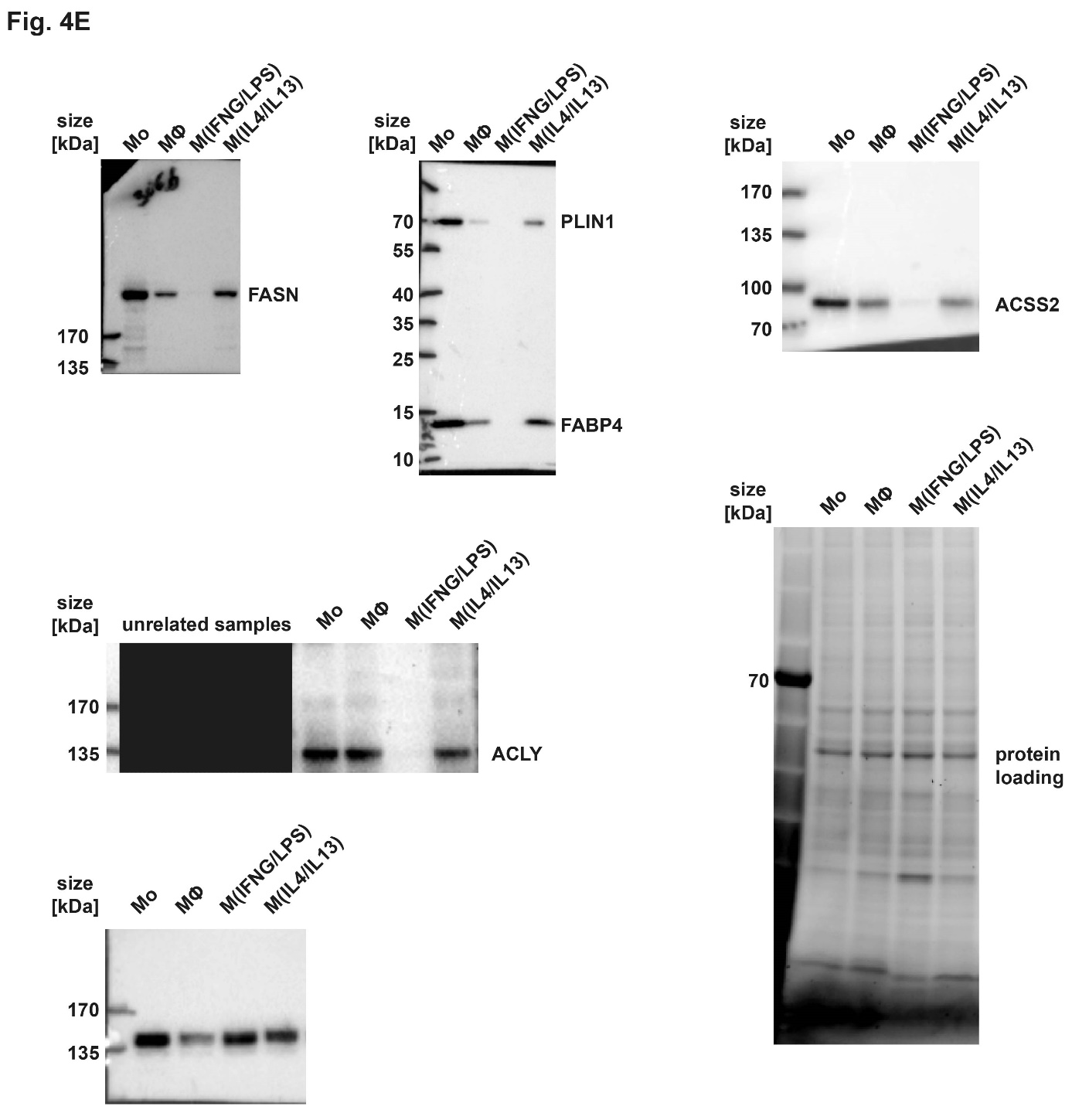
**

**Full-length unedited images for Fig. 5A**


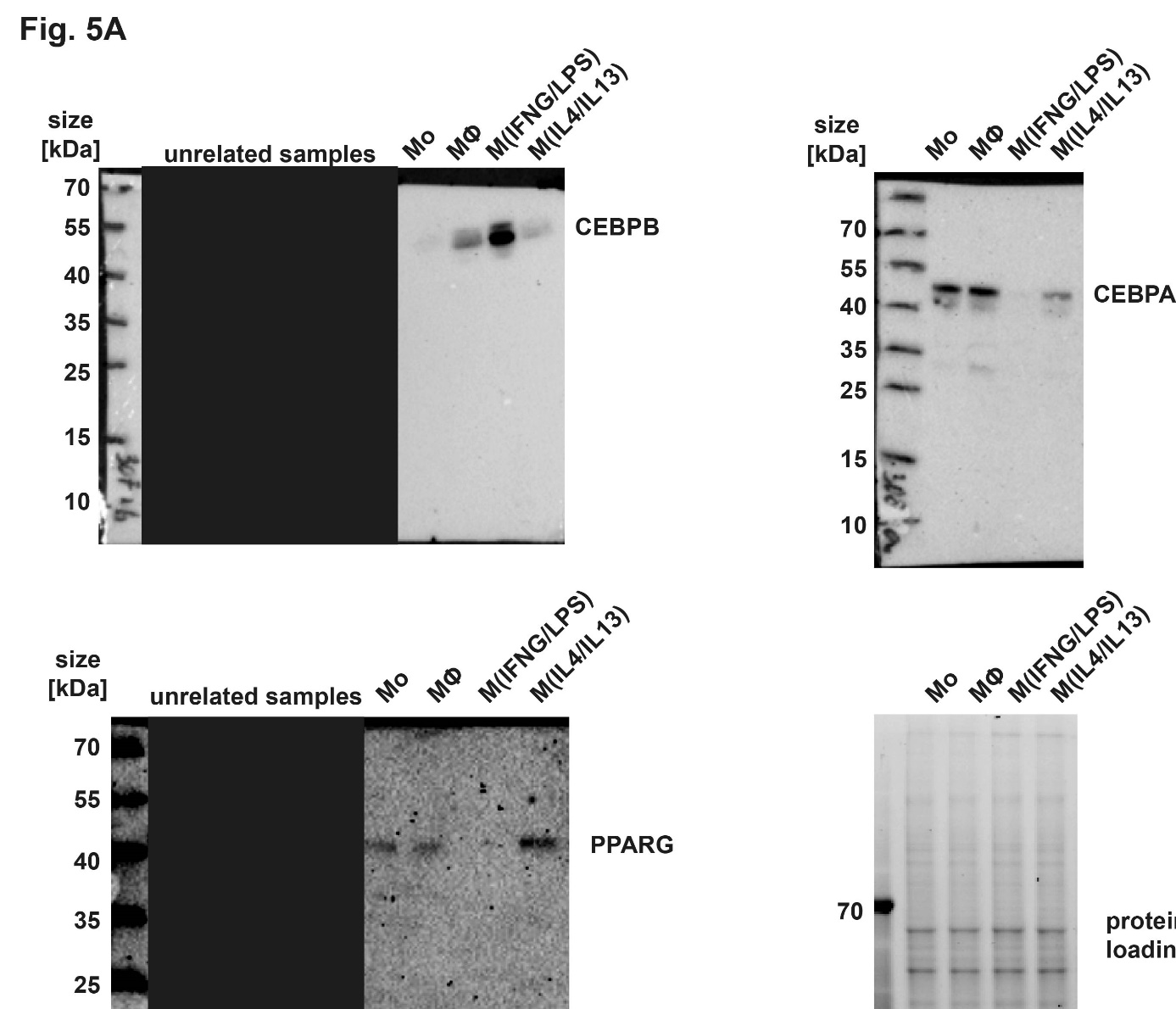


**Full-length unedited images for Fig. 5C**

**
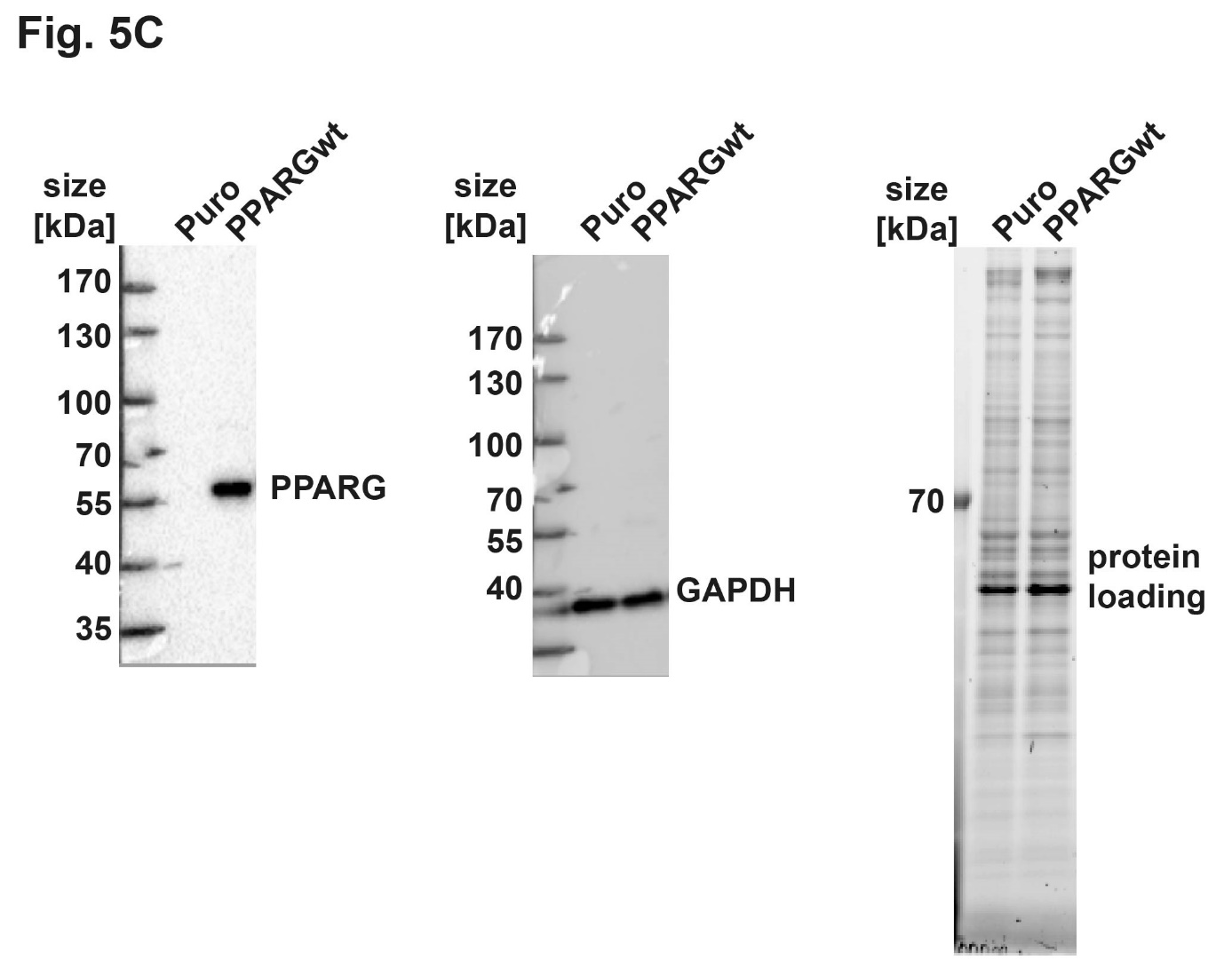
**

**Full-length unedited images for Fig. 5D**


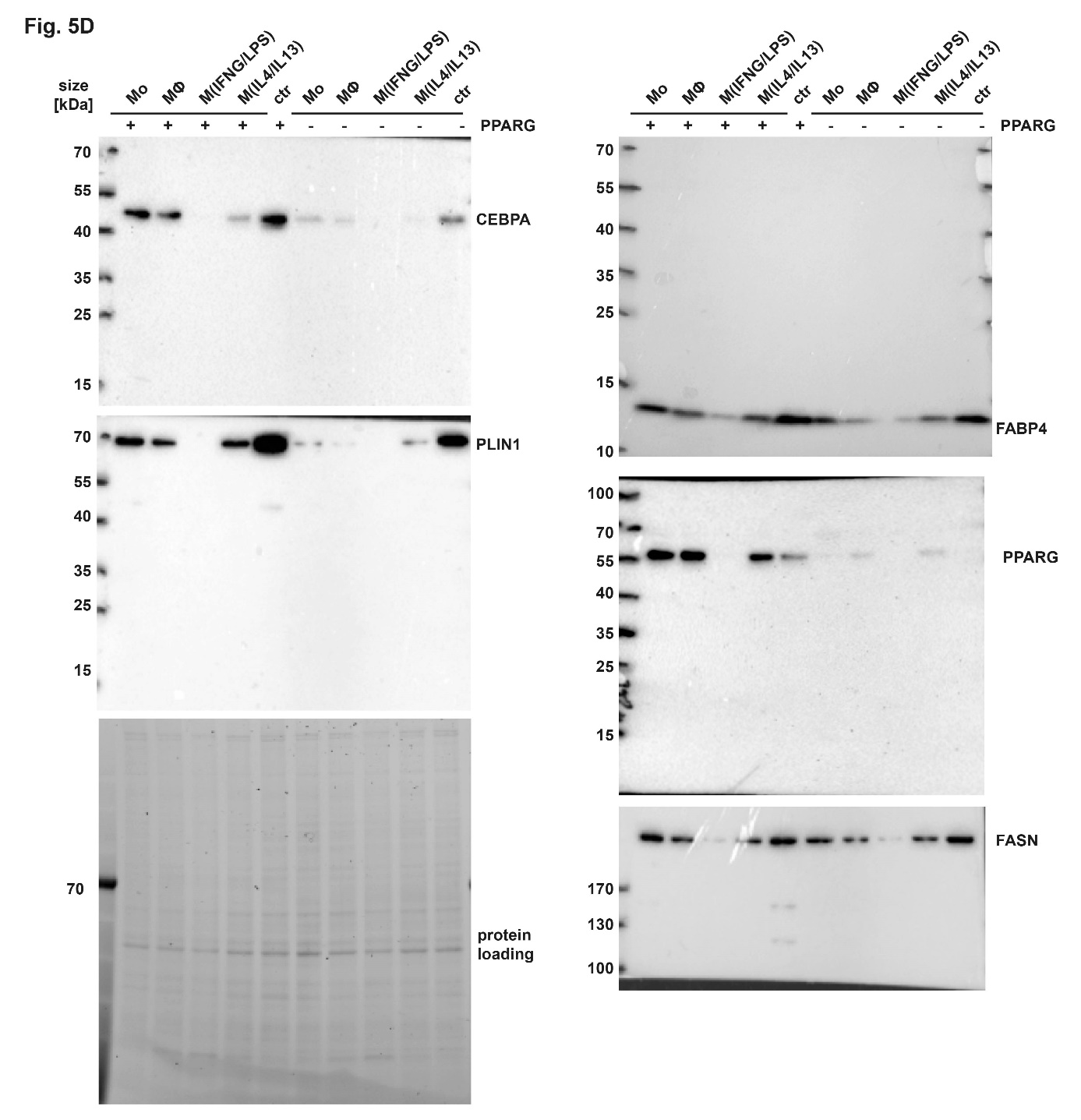


**Full-length unedited images for Fig. 6C**

**
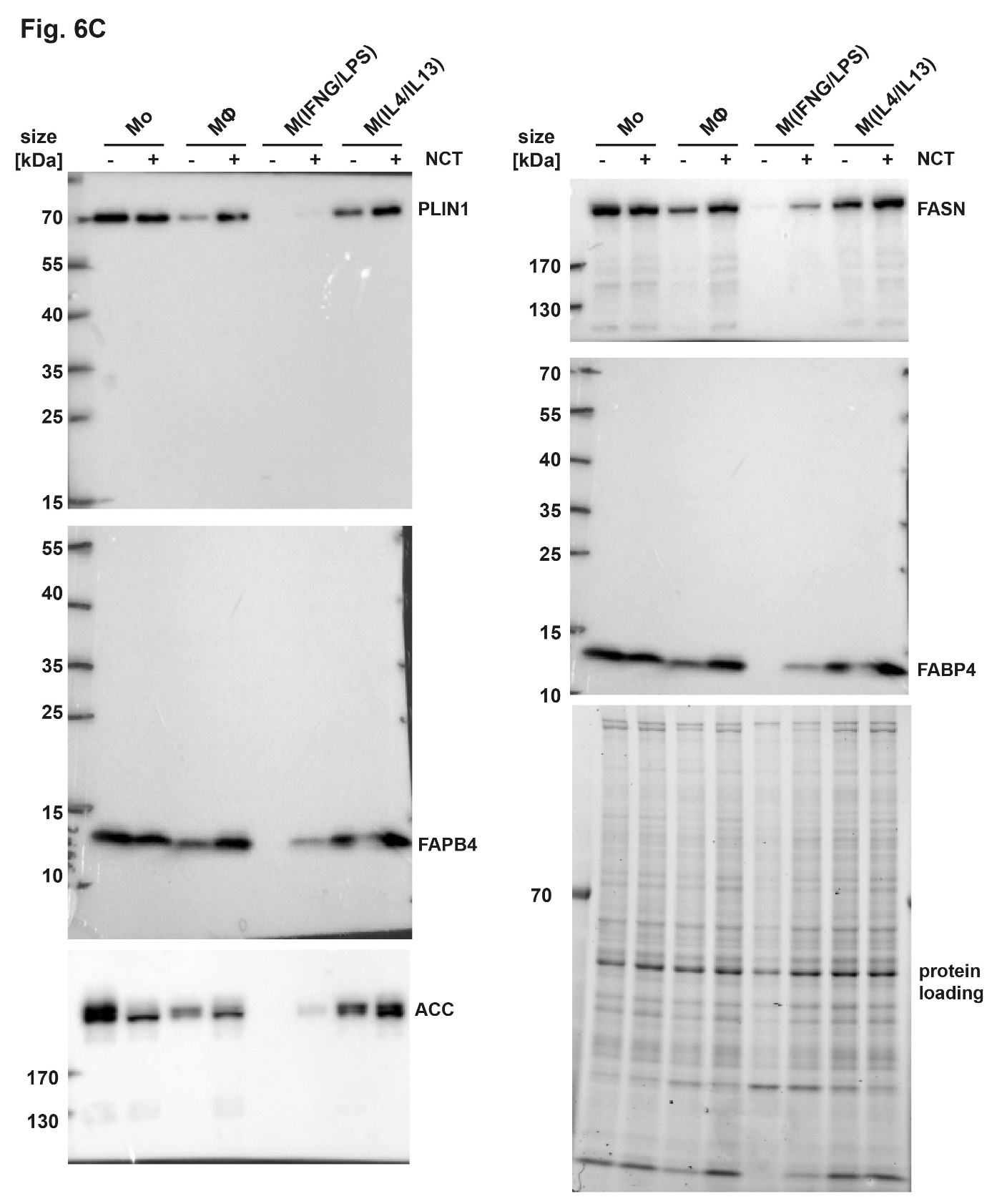
**
